# Distinguishing shear and tensile myocardial wall stiffness using *ex vivo* anisotropic Magnetic Resonance Elastography

**DOI:** 10.1016/j.actbio.2025.06.031

**Published:** 2025-06-18

**Authors:** Cyril Tous, Guillaume Flé, Stanislas Rapacchi, Matthew McGarry, Philip Bayly, Keith Paulsen, Curtis L. Johnson, Elijah Van Houten

**Affiliations:** a Centre de recherche du Centre hospitalier de l’Université de Montréal, Montréal, QC H2X 0A9, Canada; b Institute of Radiology, Universitätsklinikum Erlangen, Friedrich-Alexander-Universität Erlangen-Nürnberg (FAU), Erlangen, Germany; c Université de Lausanne, Lausanne, Vaud, Switzerland; d Thayer school of engineering, Dartmouth College, Hanover NH 03755, USA; e Department of Mechanical Engineering & Materials Science, Washington University in St Louis, St. Louis MO 63130-4899, USA; f Dartmouth-Hitchcock Medical Center, Lebanon NH 03756, USA; g Department of Biomedical Engineering, University of Delaware, Newark DE 19716, USA; h Department of Mechanical Engineering, Université de Sherbrooke, poste 62157, 2500, boul. de l’Université, Faculté de génie mécanique, Sherbrooke, QC J1K 2R1, Canada

**Keywords:** Shear, Young’s modulus, Deformation modes, Myocardial stiffness, Tensile

## Abstract

The organized myofiber structure within the myocardium indicates its mechanical anisotropy. By projecting the MR Elastography (MRE) stiffness matrix along either the myocardial *fiber* or *sheet* orientations determined by Diffusion Tensor Imaging (DTI), anisotropic MRE (aMRE) maps axial and transverse shear and Young’s moduli into three *tensile* and six *shear* deformation modes. Ten healthy *ex vivo* swine hearts were imaged three times at 3T using MRE and DTI sequences. aMRE results showed a within-subject coefficient of variation at 19 % for the fiber model and 28 % for the sheet model across specimens and metrics, with coefficients lower than 15 % for seven of the ten specimens across models. Repeatability coefficient of ±0.5 kPa for Young’s moduli and ±0.17 kPa for shear’s moduli, demonstrating repeatability within the 95 % agreement limit. Isotropic MRE underestimated stiffnesses by 31 % compared to aMRE, where anisotropic moduli aligned more closely with prior finite element studies and some mechanical loading tests. The myocardium’s anisotropic elasticity reflects with its helicoidal myofiber microstructure, with mid-wall circumferential fibers requiring twice the force to deform as longitudinal fibers at the epicardium or endocardium. At the mid-wall, fiber model values were $\mu_{\text{ax}}=1.9\pm 0.1\;\text{kPa},\;{\;\mu }_{\text{tra}}=1.3\pm 0.1\;\text{kPa}$, E_ax_ = 5.6 ± 0.4 kPa, and E_tra_ = 3.8 ± 0.3 kPa. Identified deformation modes included: (FF), (NN), (FF or SS), (NN or SS), (SN or NS), (FN or FS), (SF or FS), and (SN or NF), where N is normal to both fiber (F) and sheet (S) orientations. By aligning elasticity matrices more accurately with myocardial architecture than isotropic MRE, aMRE was able to reliably measure shear and Young’s moduli in *ex vivo* swine hearts. These mappings of deformation modes may bring myocardial stiffness assessment closer to clinical application, providing a foundation for a non-invasive methodology capable of true mechanical characterization of the cardiac wall using MR imaging.

**Statement of significance:**

The myocardium’s anisotropic elasticity, due to its helicoidal myofiber structure, is revealed through anisotropic MR elastography, using fiber and sheet elastic models. Mid-wall circumferential fibers require twice the force to deform equally compared to epicardial or endocardial fibers. Characterizing shear and Young’s moduli across cardiac modes offers noninvasive measures of ventricular compliance, comparable to pressure-volume relationships. This could enhance early diagnosis of “stiff heart syndrome” and clarify its underlying mechanisms. Additionally, it aids understanding of myocardial pathologies, including amyloidosis, hypertrophic and dilated cardiomyopathies, and ischemic damage. By characterizing tensile and shear interactions, it may inform diagnosis and treatment of conduction issues and arrhythmia, where tissue has lost its normal mechanical behavior, while patient-specific models could optimize surgical and therapeutic outcomes.

## 1. Introduction

The mechanical and electrical function of the heart is influenced by passive myocardial stiffness in the atria and ventricles [1,2]. Elevated passive stiffness in the myocardium is associated with dysfunction in both systolic and diastolic phases [3]. Routine assessment of myocardial viscoelasticity, both regionally and globally, could improve clinicians’ ability to evaluate the underlying mechanisms of heart failure. For example, measuring the extent and severity of stiffened wall segments, as well as biomechanical differences across necrotic, border, stunned, hibernating, and fibrotic zones may provide valuable insight into disease progression and the risk of increased filling pressures. The standard method for assessing *‘chamber stiffness’ in vivo* involves analyzing pressure-volume loops, particularly the end-diastolic pressure-volume relationship (EDPVR). Lately, machine learning methods have been successful in speeding up patient-specific *in vivo* estimation of myocardial stiffness from EDPVR [4,5]. However, the process of measuring pressure-volume loops across a broad range of preloads to construct the EDPVR is challenging and time-consuming [6] and yields a single parameter for an entire ventricle. Spatially resolved anisotropic mechanical property images measured with cardiac MR elastography (MRE) would help to map the axial and transverse shear and Young’s moduli and assess their correspondence with the three tensile and six shear deformation modes that characterize myocardial mechanics over the cardiac cycle. This approach would enhance our understanding of how changes in stiffness relate to myocardial structure, effectively linking myocardial function and structural organization through these specific deformation modes.

**Fig. 1.**
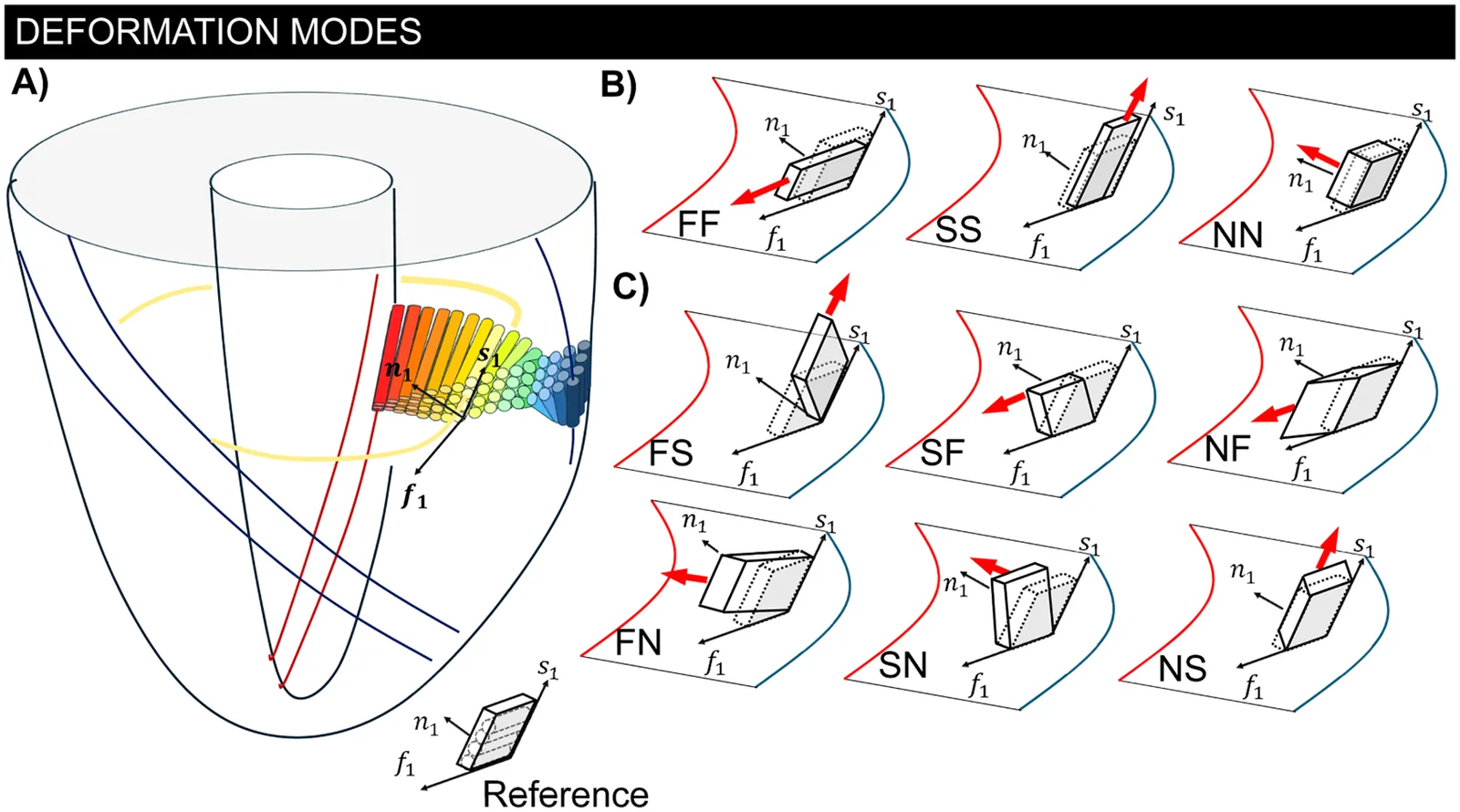
Myocardial architecture of the left ventricle derived from diffusion tensor imaging (DTI) (**A**) showing the helical architecture of the fiber at the endocardium (red), mid-wall (yellow) and epicardium (blue). Mechanical properties in the local reference frame can be decomposed into three tensile deformation modes (FF, SS, NN in **B**) and six shear deformation modes (FS, SF, NF, FN, SN, and NS in **C**).

To date, MRE has only been used to estimate the mean values of the shear storage modulus of the myocardium, without accounting for its anisotropic structure [7–13]. The myocardium consists of fiber layers arranged in a distinct helical pattern from the endocardium to the epicardium (Fig. 1-A). Fibers are aggregated transmurally by a collagen matrix to form laminar sheetlets separated by cleavage planes [14]. MR diffusion tensor imaging (DTI) can map myofiber orientation as it corresponds to the primary direction of Brownian motion for water molecules, whereas the inclination of the sheetlets aligns with the second diffusion tensor eigenvector. The direction normal to the sheetlet plane, which also corresponds to the direction of sheetlet stacking, aligns with the third diffusion tensor eigenvector [15]. The viscoelastic nature of the myocardium can be decomposed into its three components within this DTI reference frame: along the fiber, F, along the second orientation in the sheetlet plane, S, and perpendicular to the sheetlet plane, N. Each of these orientations is characterized by specific shear and Young’s moduli describing stiffness. Thus, a 3 × 3 tensor of combined tensile and shear deformation modes is required to comprehensively characterize the myocardium: [FF, FS, FN; SS, SF, SN; NN, NF, NS]. The directional dependence of the myocardium’s non-linear viscoelastic shear properties in the passive state has already been observed as a function of increasing strain [16,17], where the tensile deformation modes (Fig. 1-B) and shear deformation modes (Fig. 1-C) could be identified.

A recent MRE study in the brain, where axonal fiber tracts give rise to similar anisotropic mechanical behavior, has shown that isotropic MRE reconstruction can lead to property value discrepancies of up to 30 % [18], highlighting the need for anisotropic modeling for accurate property estimates. We have recently introduced a finite element (FE)-based anisotropic MR elastography (aMRE) method which leverages DTI to define the orientation of mechanical symmetry for a transversely-isotropic anisotropy model. The nonlinear optimization algorithm iteratively updates the anisotropic mechanical property distribution to fit the predicted displacements from the FE model to the measured displacements [19–21].

In this study, we aim to non-invasively quantify shear and tensile moduli of the heart using aMRE and assess the repeatability of these values across three repeated MRE scans for each of the ten *ex vivo* hearts. The moduli analyzed include the axial and transverse shear moduli $\left(\mu_{\text{ax}},\;\mu_{\text{tra}}\right)$, the axial and transverse Young’s moduli $\left(E_{\text{ax}},\;E_{\text{tra}}\right)$, and the shear and tensile anisotropy measures (ϕ,ζ).

**Fig. 2.**
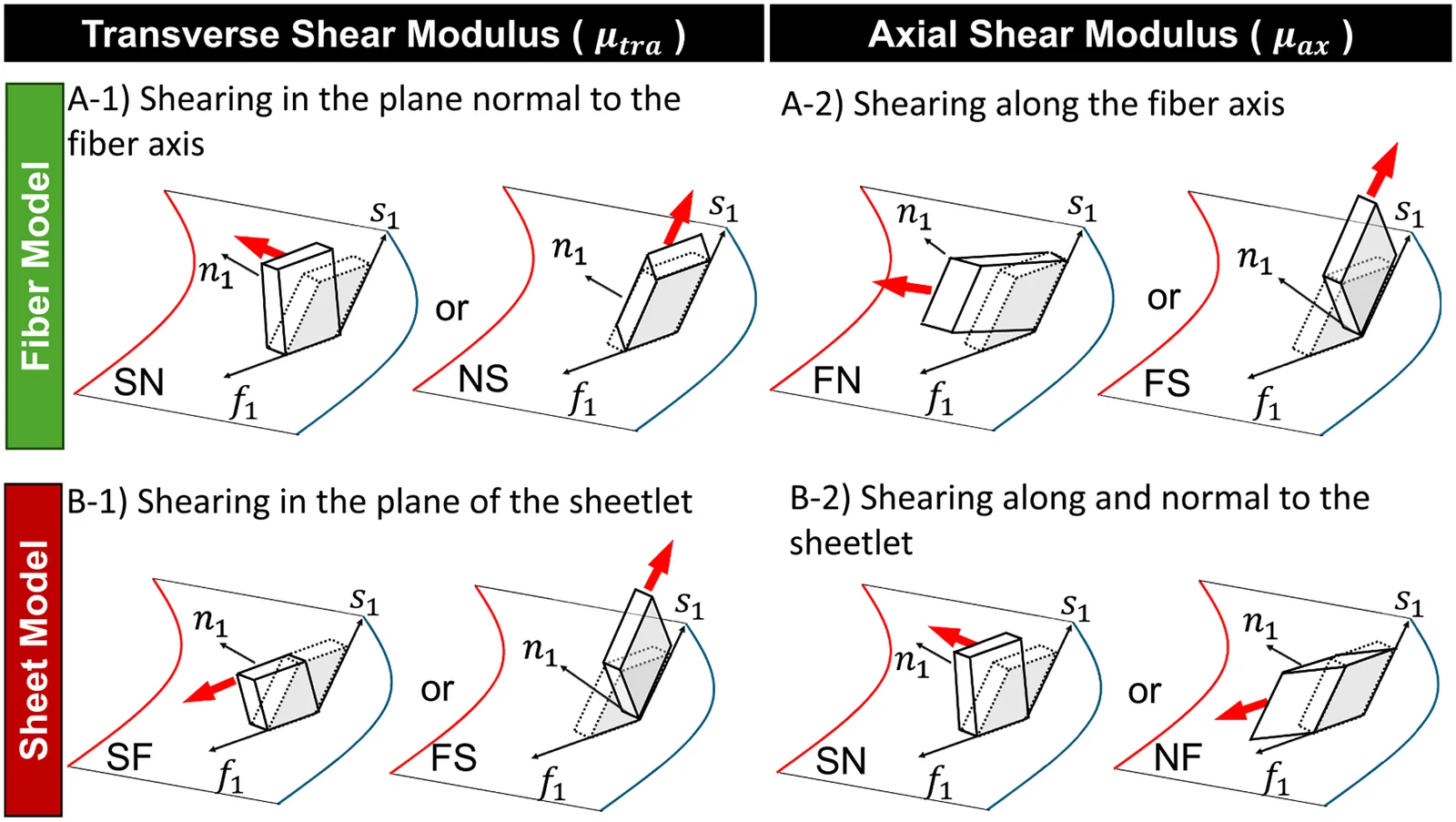
The fiber model (A) and sheet model (B) with their respective transverse ($\mu_{\text{tra}}$ in (1)) and axial ($\mu_{\text{ax}}$ in (2)) shear moduli and corresponding deformation modes. Colored arrows indicate the direction of shearing. Sheets are represented as rectangular prisms. There are three axes: the fiber axis $\left(f_{1}\right)$, the sheet axis $\left(s_{1}\right)$, and normal to the sheet plane $\left(n_{1}\right)$.

**Fig. 3.**
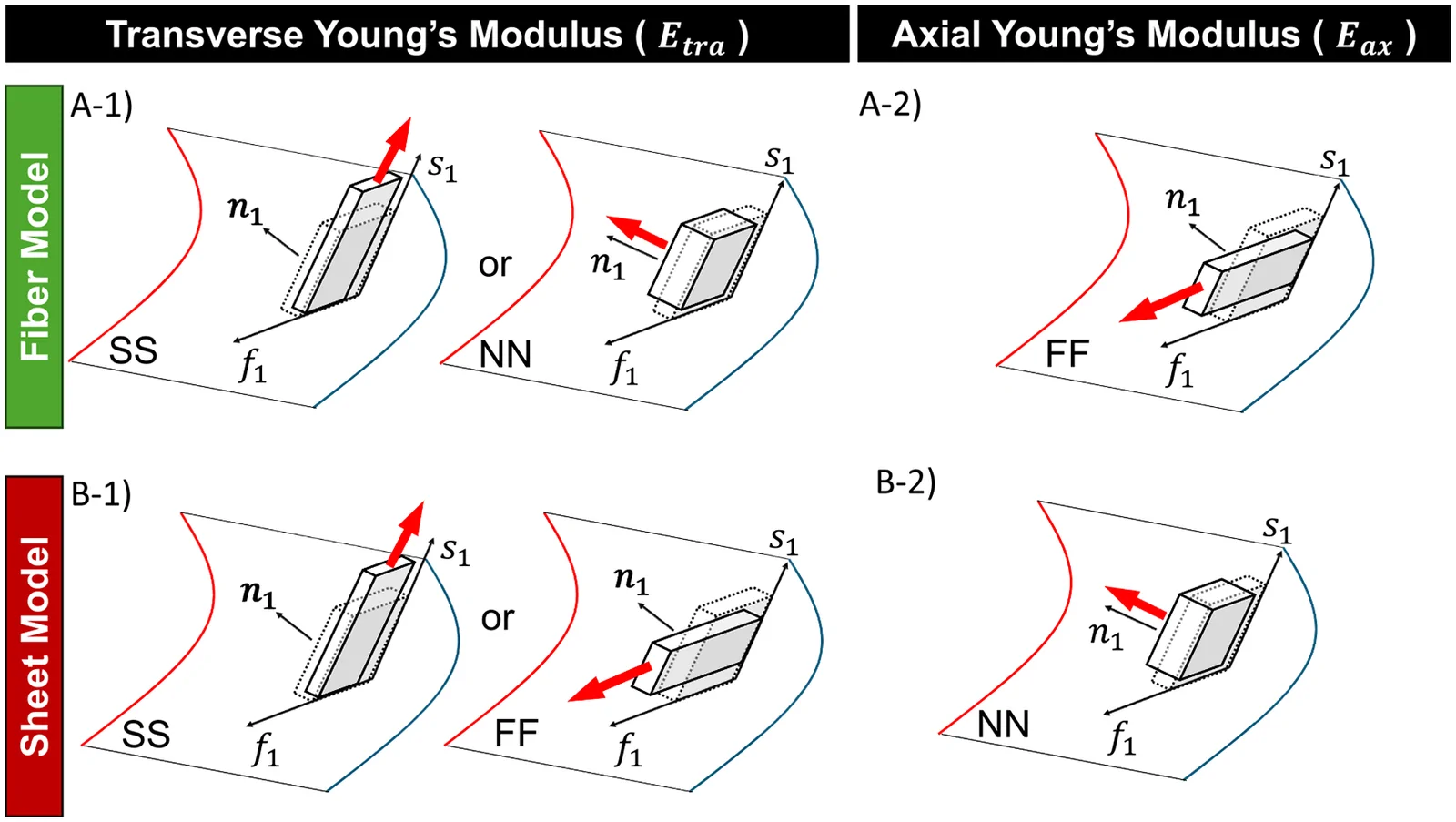
The fiber model (A) and sheet model (B) with their respective transverse ($E_{\text{tra}}$ in (1)) and axial ($E_{\text{ax}}$ in (2)) Young’s moduli and corresponding deformation modes. Colored arrows indicate the direction of stretching, where sheets are represented as rectangular prisms. Tensile deformation modes are shown relative to the fiber (F), sheet (S) and normal-to-sheet (N) planes, with red arrows indicating the direction of stretching. FF, SS, and NN correspond to the conventional stretching directions identified in mechanical loading studies (*e.g*. hysteresis curves).

In aMRE, the elasticity matrix of the transversely isotropic material can be rotated to align with either the primary fiber axis (first eigenvector of the diffusion tensor), leading to an anisotropy description referred to here as the “*fiber model*”, or the axis normal to the sheet plane (third eigenvector of the diffusion tensor), referred to here as the “*sheet model”*. The axis of rotation determines which pair of cardiac deformation modes (Fig. 1-B and C) corresponds to the calculated modulus with aMRE (Figs. 2 and 3). For example, in the fiber model, $\mu_{\text{tra}}$ corresponds to shear deformation modes like SN or NS, which are transverse to the fiber direction, while $\mu_{\text{ax}}$ corresponds to shear deformation modes like FS (SF) or FN (NF), which involve shearing within or along the principal fiber direction.

## 2. Materials and methods

### 2.1. MRI acquisitions

Ten freshly excised healthy *ex vivo* swine hearts [N=10 (5 females and 5 males), weight: 35 to 50 kg, age: 3 to 7 months] (Ferme Triporc Inc., Quebec, Canada) were scanned within one hour after euthanasia with 540 mg/kg pentobarbital sodium. As a result, the excised hearts in this study were in a passive state, allowing for the characterization of the myocardium’s nonlinear viscoelastic shear properties. After euthanasia, ATP levels began decreasing and progressive tissue stiffening of the myocardium limited the duration of our study [22]. This phenomenon, called rigor mortis, begins approximately two to four hours after death and fully develops after 12 h [23]. In our study, no chemicals were used to slow down decomposition or preserve the tissue. Although formaldehyde prevents tissue decay, it rapidly increases tissue stiffness [24]. All freshly excised specimens were inspected by a veterinarian, deemed healthy, and showed no ischemic regions. They were scanned immediately after euthanasia for up to one hour. Scanning was performed with a 3.0T MRI unit (Magnetom Skyra, Siemens Healthineers, Erlangen, Germany). Each heart was placed on a 32-channel spine coil and the active driver (Resoundant, Rochester, MN, USA) was in the MRI control room and the passive driver was placed directly on the anterior basal side of the heart to deliver vibrations to the tissue and was covered with an 18-channel Tim chest coil.

All animal studies adhered to the National Institutes of Health (NIH) Guide for the Use and Care of Animals and were conducted in accordance with the Canadian Council on Animal Care. The study was approved by the CHUM (Centre Hospitalier de l’Universite de Montréal) Research Center’s Institutional Animal Care Committee (C17014GSp, # IM17014SKp).

Mechanical vibrations during MRE were induced at 100 Hz and encoded in a spin-echo EPI MRE sequence [25]. Imaging parameters included: Field of View= 340 × 340 mm^2^; TR/TE = 540/109 ms; Bandwidth = 1730 Hz/pixel, three slices and 2 × 2 × 2 mm^3^ isotropic voxel; motion encoding gradient amplitude = 40 mT/m. The images were acquired below the heart valves, in a basal plane where the septum is thickest. Displacement measurements were performed in the three orthogonal spatial directions (x, y, z), with positive and negative motion encoding to generate a phase difference image free from unwanted background phase signal. The acquisition of four phase offsets, between the mechanical excitation and the motion-sensitizing gradients, equally spaced across the harmonic cycle, resulted in a total of 24 measurements.

MRE data were acquired consecutively three times to assess scan-wise repeatability. Colocalized DTI data, with resolution and field of view matched to the MRE acquisition, were also acquired with the following imaging parameters according to a previous study on *ex vivo* hearts [26]: spin echo EPI, 32 diffusion-encoding gradients at $b=557\;\text{s}/{\text{mm}}^{2}$ (3 averages); TR/TE = 1500/70 ms. Increasing the number of averages enhances the characterization of the primary eigenvector, while acquiring more diffusion directions improves the differentiation between eigenvectors. A minimum of 30 diffusion directions is required for a robust estimation of the diffusion tensor and mean diffusivity. However, acquiring an excessive number of averages does not necessarily yield additional information about the primary eigenvector [27–30].

In this study, we limited the spatial coverage to three slices for several reasons. (1) Acquiring more DTI and MRE slices would have extended scan time, increasing the risk of rigor mortis affecting myocardial stiffness. We minimized this effect and ensured physiological accuracy while also measuring scan-wise repeatability by repeating the imaging protocol three times. (2) It is standard practice to only image three slices *in vivo* cardiac DTI. As mentioned previously, there is a necessary trade-off between spatial coverage, b-values, signal-to-noise ratio (SNR), the number of diffusion directions acquired, and averaging. (3) We aimed to establish a clinically applicable framework that can be implemented on 3T clinical MRI scanners for human patients with cardiac conditions, using realistic MR sequences that align with reasonable clinical scan time, as done in other studies [31–34].

### 2.2. Theory

In MRE, the displacement field generated by the external passive driver can be described by the time-harmonic elastic equilibrium equation:

(1) $$\nabla \cdot \sigma =-\rho \omega^{2}u$$

Which, in the case of an isotropic material model, can be expanded to:

(2) $$\nabla \cdot \left(\mu \left(\nabla u+\nabla u^{T}\right)\right)+\nabla \left(\lambda \nabla \cdot u\right)=-\rho \omega^{2}u,$$

where u is the displacement field measured in **[m]** at angular frequency ω in **[rad/s]**, σ is the rank 2 stress tensor; μ is the shear modulus in **[Pa]**, $\mu =Re\left[\mu \right]+i\;Im[\mu ]$, with Re[μ] the storage modulus, Im[μ] the loss modulus and i the imaginary constant i=sqrt(-1); λ is a material modulus **[Pa]** related to volume compressibility; and ρ is the mass-density of the material in **[kg/m**^**3**^].

The anisotropic mechanical properties of the heart were reconstructed using the Nonlinear Inversion (NLI) MRE method formulated with a finite element (FE) model of a nearly incompressible, transversely isotropic material (TI-NLI) [19,20,35,36]. The TI-NLI algorithm accurately estimates spatial contrast in anisotropic properties, as verified in anisotropic MRE phantoms [37]. The symmetry axis of the transversely isotropic material at each voxel location is rotated based on DTI data to align either with the primary fiber axis (first eigenvector of the diffusion tensor), a configuration defined here as the “*fiber model*”, or with the third eigenvector of the diffusion tensor, aligned with the axis normal to the sheet plane, a configuration defined here as the “*sheet model*”. The constitutive equation for this material, defining the relationship between the **stress** σ and the **strain** ϵ, assuming fibers are oriented along the direction $x_{1}$, (*i.e*. the fiber model), is given by the sparse symmetric tensor:

(3) $$\left\{\begin{array}{l} \sigma_{11}\\ \sigma_{22}\\ \sigma_{33}\\ \sigma_{12}\\ \sigma_{23}\\ \sigma_{13} \end{array}\right\}=\left[\begin{array}{cccccc} {\text{c}}_{11} & {\text{c}}_{12} & {\text{c}}_{13} & 0 & 0 & 0\\ {\text{c}}_{21} & {\text{c}}_{22} & {\text{c}}_{23} & 0 & 0 & 0\\ {\text{c}}_{31} & {\text{c}}_{32} & {\text{c}}_{33} & 0 & 0 & 0\\ 0 & 0 & 0 & {\text{c}}_{44} & 0 & 0\\ 0 & 0 & 0 & 0 & {\text{c}}_{55} & 0\\ 0 & 0 & 0 & 0 & 0 & {\text{c}}_{66} \end{array}\right]\left\{\begin{array}{c} \epsilon_{11}\\ \epsilon_{22}\\ \epsilon_{33}\\ 2\epsilon_{12}\\ 2\epsilon_{23}\\ 2\epsilon_{13} \end{array}\right\},$$

with the components of the 6 × 6 elasticity matrix, [C], given by

(4) $$c_{11}=\kappa +\frac{4}{3}\mu_{\text{tra}}\left(1+\frac{4}{3}\zeta \right),\;c_{22}=c_{33}=\kappa +\frac{4}{3}\mu_{\text{tra}}\left(1+\frac{1}{3}\zeta \right),\;c_{44}=c_{66}=\mu_{\text{tra}}(1+\phi ),\;c_{12}=c_{13}=c_{21}=c_{31}=\kappa -\frac{2}{3}\mu_{\text{tra}}\left(1+\frac{4}{3}\zeta \right),\;c_{32}=c_{23}=\kappa -\frac{2}{3}\mu_{\text{tra}}\left(1-\frac{2}{3}\zeta \right),\;c_{55}=\mu_{\text{tra}}$$

from (Eq (4)), where, when considering the fiber model, $\mu_{\text{tra}}$ is the shear modulus in the plane normal to the fiber axis, ϕ is the shear anisotropy, $\phi =\frac{\mu_{\text{ax}}}{\mu_{\text{tra}}}-1,\;\zeta$ is the tensile anisotropy, $\zeta =\frac{E_{\text{ax}}}{E_{\text{tra}}}-1$, and κ is the isotropic bulk modulus.

$\left(c_{11},c_{22},c_{33}\right)$ represent the normal stress responses and are related to the isotropic bulk modulus and the directional moduli.

$\left(c_{44},c_{55},c_{66}\right)$ represent shear moduli in planes normal to the principal axes and define the level of shear stress due to shear deformation.

$\left(c_{12},c_{13},c_{23}\right)$ are often zero in ideal isotropic materials but non-zero in anisotropic materials, indicating coupling between different strain components.

From (Eq (4)), it can be seen that the two axial shear moduli, $\mu_{\text{ax}}$, corresponding to shearing along the fiber axis in the fiber model, are given by $c_{44}$ and $c_{66}$, while the transverse shear modulus, $\mu_{\text{tra}}$, corresponding to shearing around the fiber axis in the fiber model (*i.e*. no fiber influence), is given by $c_{55}$. As a result, we obtain:

(5) $$E_{\text{ax}}=E_{\text{tra}}(1+\zeta )=3\mu_{\text{tra}}(1+\zeta )$$

These alignments and the understanding of C’s structure are essential to accurately interpret the MRE data and to these measurements to the underlying biomechanical properties of the heart, allowing the eventual diagnosis and study of various cardiac diseases.

### 2.3. The fiber model, sheet model and isotropic incompressible model

In the fiber model, the shear deformation mode pair (SN, NS) represents the resistance to shearing perpendicular to the fiber axis $\left(\mu_{\text{tra}}\right)$. Conversely, the shear deformation mode pair (FN, FS), is obtained by shearing along the fiber axis $\left(\mu_{\text{ax}}\right)$ (Fig. 2-A). Therefore, in the fiber model, the assumption is that there is a dominant fiber orientation, and everything else is considered “perpendicular” to the fibers.

Alternatively, the sheet model can distinguish between the shear deformation mode pairs (SF, FS), represented by $\mu_{\text{tra}}$, and (SN, NF), represented by $\mu_{\text{ax}}$ (Fig. 2-B). Thus, in the **sheet model**, the assumption is that within the sheet plane, the properties remain uniform in every direction, while a different set of properties govern deformations with components normal to the sheet plane.

The respective tensile deformation modes for the two anisotropy models are: (SS, NN), represented by $E_{\text{tra}}$, and (FF), represented by $E_{\text{ax}}$ for the fiber model (Fig. 3-A), and (SS, FF), represented by $E_{\text{tra}}$, and (NN), represented by $E_{\text{ax}}$ for the sheet model (Fig. 3-B). More detailed sketches are presented in the Supplementary Materials (Figure S.8 and Figure S.9)

In *isotropic* MRE models, elastic properties are considered uniform in all directions (*i.e*., no distinction between the axial and transverse components, $E_{\text{ax}}=E_{\text{tra}}={\;3\mu }_{\text{tra}}={\;3\mu }_{\text{ax}}$), which has been shown to result in the inaccurate characterization of mechanically anisotropic materials [18].

### 2.4. MRE reconstruction; the subzone-based nonlinear inversion method

Van Houten *et al*. have proposed an FE-based iterative inversion technique using overlapping subzones that identify tissue mechanical properties at the same spatial resolution as an MR voxel [36]. Subzones are modeled as parallelepipeds whose size is defined by the user so that they contain at least half a shear wavelength [38]. They are then randomly distributed within the bounded domain of the displacement images, at the start of each global iteration. Overlapping subzones are processed in parallel for global error minimization within an acceptable computation time.

High-performance computing resources from Calcul Québec (calculquebec.ca) and Digital Research Alliance of Canada (alliance.can.ca) were used to perform the TI-NLI MRE reconstruction. The mechanical property distributions within each subzone are determined by minimizing a cost function between the MR measured tissue displacement and the displacements calculated from FE models of the tissue (Eq. (1)), based on the currently estimated mechanical property distribution within that subzone.

**Fig. 4.**
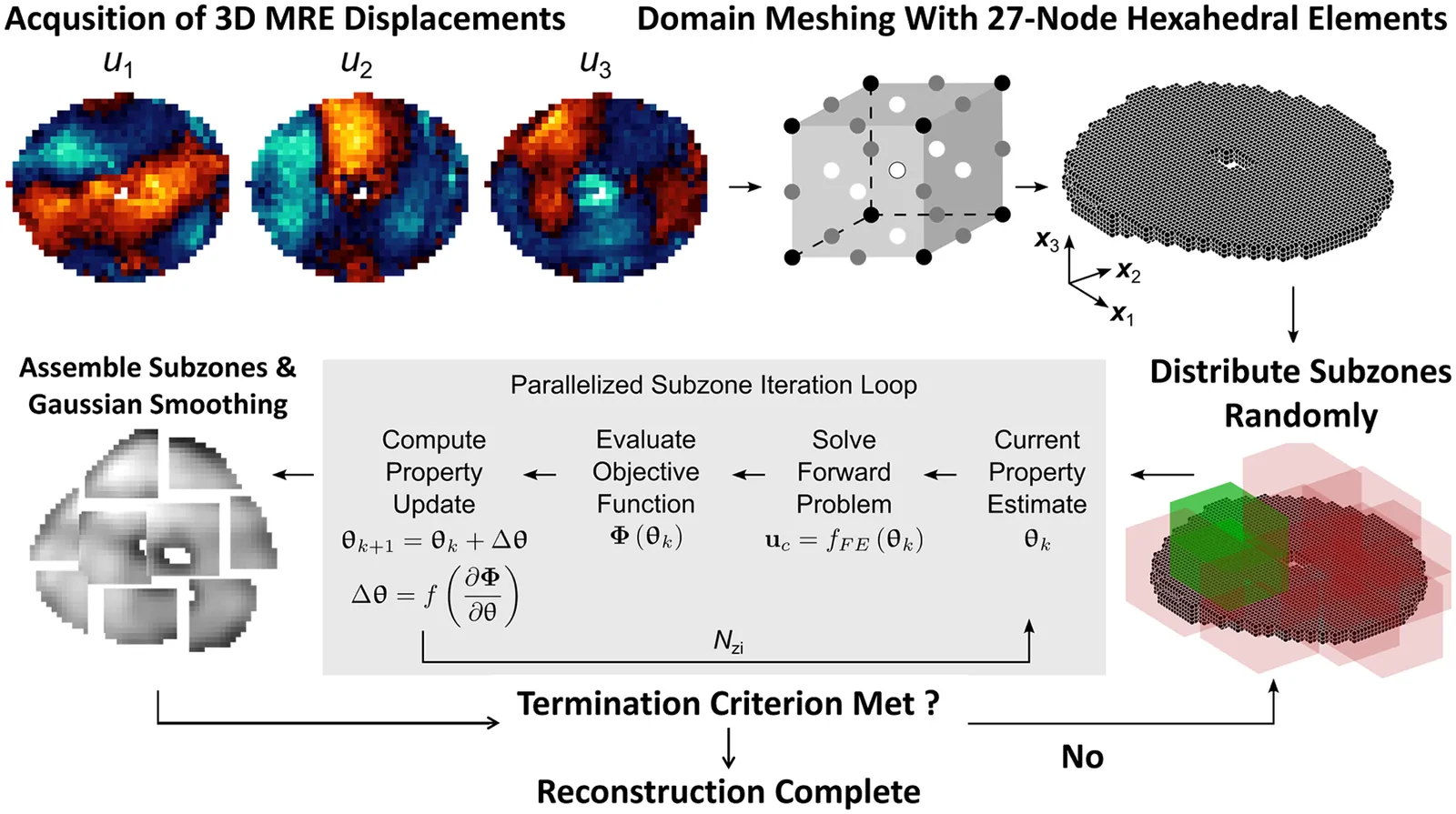
Workflow of the subzone-based NLI reconstruction method using an FE-based formulation of the nearly incompressible, transversely isotropic material model.

For *in vivo* MRE, the density of the tissue (ρ) is typically predefined as the density of water, $\rho =1\;g/{\text{cm}}^{3}$. Likewise, the bulk modulus, $\kappa =\frac{2\;Re[\mu ](1+\nu )}{3(1-2\nu )}$, was fixed at κ=9GPa, modelling a nearly incompressible tissue with Poisson’s ratio, ν=0.4999998, and a shear modulus consistent with soft tissue, Re[μ]=2kPa. The bulk modulus has a negligible effect in MRE as long as κ>100μ. More information on the initial estimated parameters used in the gradient descent optimization is provided in Table S.1. To optimize the convergence of anisotropy estimates (ζ and ϕ) during the TI-NLI algorithm for reconstruction, the property mesh resolution was coarser in the first iterations (5 mm from iterations 1 to 10) and gradually refined in the subsequent iterations (2 mm from iterations 10 to 70; 1 mm from iteration 70 to 300) [39] (Fig. 4). The shear modulus (μ) was kept at 1 mm resolution for the entire inversion. This approach divides the global inversion into a set of smaller scale inversions and reduces the risk of failing to correctly characterize a particular region due to its successive inclusion in different subzones.

The transversely isotropic nonlinear inversion (TI-NLI) method presented by McGarry et al. [19] was used in this study to investigate anisotropic effects in the heart. Eckstein et al. validated the TI-NLI method’s ability to characterize both isotropic and anisotropic material regions in anisotropic MRE phantoms [37]. The small-deformation linear elasticity model used by the TI-NLI algorithm assumes that antagonistic shearing directions are equivalent, leading to the following mode equivalences: SF = FS, NS = SN, and NF = FN.

Suitability of the MRE displacement data for the nonlinear inversion was assessed using the Octahedral Shear Strain Signal-to-Noise Ratio (OSS-SNR) [40]. Using this approach, we non-invasively obtained mappings of the following cardiac deformation modes from the NLI-MRE subzone method and the two proposed models: (SN or NS), (FN or FS), (SF or FS), (SN or NF), (FF), (NN or SS), (NN) and (FF or SS).

The proposed TI-NLI aMRE model cannot independently identify each of the six shear deformation modes and three tensile deformation modes. Applying the axis of symmetry to the second sheetlet population (second eigenvector) yields the following mode combinations:

$$\mu_{\text{ax}}=\left(\text{SN}\;\text{or}\;\text{FS}\right),$$

$$\mu_{\text{tra}}=\left(\text{FN}\;\text{or}\;NF\right),$$

$$E_{\text{ax}}=\left(\text{SS}\right),$$

$$E_{\text{tra}}=\left(\text{FF}\;\text{or}\;\text{NN}\right),$$

which facilitates the isolation of yet other cardiac deformation modes. It is important to note that each of these shear deformation modes represents an independent deformation component. Therefore, linear combinations of these deformation modes cannot be used to generate separate descriptions of the different shear deformation modes.

Axial and transverse shear and Young’s moduli were analyzed using the transmural distance from the endocardium. Once the image was manually segmented, the radial axis was defined as the shortest distance between the endocardial wall (0–10 % depth) and the epicardial wall (90–100 % depth), following the method described by Tous et al. [26]. Finally, a variation analysis across specimens was performed to determine whether outliers, caused by rigor mortis or unknown conditions, were present.

### 2.5. Statistics

#### 2.5.1. Within-specimen coefficient of variation

Repeatability of the MRE metrics was determined with the within-specimen coefficient of variation (wCV), a relative measure expressed as a percentage, from pairwise comparisons of repeated scans (n=3), where, from [41], we have:

$$\text{SD}=\sqrt{\frac{\sum {\left({\text{x}}_{1}-{\text{x}}_{2}\right)}^{2}}{2\text{n}}},$$

$$w\text{CV}\left(\%\right)=100\times \frac{\text{SD}}{\frac{\sum \left({\text{x}}_{1}+{\text{x}}_{2}\right)}{2\text{n}}},$$

with $x_{1}$ and $x_{2}$ are the pair of measurements for a total of n measurements, and SD is the standard deviation.

#### 2.5.2. Repeatability coefficient

The repeatability coefficient (RC), a direct measure of absolute repeatability, provides a range within which repeated measurements of the same specimen are expected to fall 95 % of the time.

RC=1.96×wSD

where wSD is the within-subject standard deviation, a measure of the variability of repeated measurements within the same subject, with

$$\text{wSD}=\frac{\text{LOA}}{1.96\times \sqrt{2}}$$

where LOA are the 95 % limits of agreement in a Bland-Altman analysis, as defined below.

#### 2.5.3. Relative mean absolute difference

Relative mean absolute difference was used to check consistency between measured aMRE metrics across the fiber and sheet models.

The relative mean absolute difference (RMD) was calculated as RMD=MD/AM where the absolute mean difference MD is $\left\|x_{1}-{\;x}_{2}\right\|$ and the arithmetic mean AM is $\frac{x_{1}+x_{2}}{2}$, with $x_{1}$ and ${\;x}_{2}$ the pair of measurements from the two different models..

#### 2.5.4. Difference between two sets of measurements from different methods

An independent two tailed t-test, t, with unequal variances (Welch’s t-test) was used with

$$t=\frac{{\text{mean}}_{1}-{\text{mean}}_{2}}{\sqrt{\frac{{\text{s}}_{1}^{2}}{n_{1}}+\frac{{\text{s}}_{2}^{2}}{n_{2}}}}$$

where ${\text{mean}}_{1}$ and ${\text{mean}}_{2}$ are the means of the two groups, $s_{1}$ and $s_{2}$ are the standard deviations, and $n_{1}$ and $n_{2}$ are the sample sizes of the groups respectively.

The degrees of freedom, df, for Welch’s t-test, which doesn’t assume equal variances, was calculated using the Welch–Satterthwaite equation:

$$df=\frac{{\left(\frac{s_{1}^{2}}{n_{1}}+\frac{s_{2}^{2}}{n_{2}}\right)}^{2}}{\frac{{\left(\frac{s_{1}^{2}}{n_{1}}\right)}^{2}}{n_{1}-1}+\frac{{\left(\frac{s_{2}^{2}}{n_{2}}\right)}^{2}}{n_{2}-1}}$$

Consequently, using T.DIST.2T from Excel (Microsoft Corporation. *Microsoft Excel*. 2016. Office16. Redmond, WA) to get the p-value, with the t-statistic and degrees of freedom, such as:

$$p_{\text{value}}\;=\;T.\text{DIST}.2T(\text{abs}(t),\;df)$$

To control for the family-wise error rate given multiple comparisons, the Holm-Bonferroni method was used. This method adjusts p-values to reduce the likelihood of type I errors (false positives). The p-values were sorted in ascending order, then were adjusted for each p-value by multiplying by the number of remaining comparisons: $p_{\text{adj}}=p\times \left(n-i\right)$ where n is the total number of tests, and i is the index of the sorted p-value. Finaly, the adjusted p-values were capped at 1.0 to ensure they remain valid probabilities. Statistical significance was defined as p<0.05.

#### 2.5.5. Bland-Altman analysis

Bland-Altman analysis was used to assess whether the mean bias was within the 95 % confidence interval (CI) of the mean difference across repeated MRE acquisitions.

## 3. Results

### 3.1. Mapping and signal-to-noise ratio

**Fig. 5.**
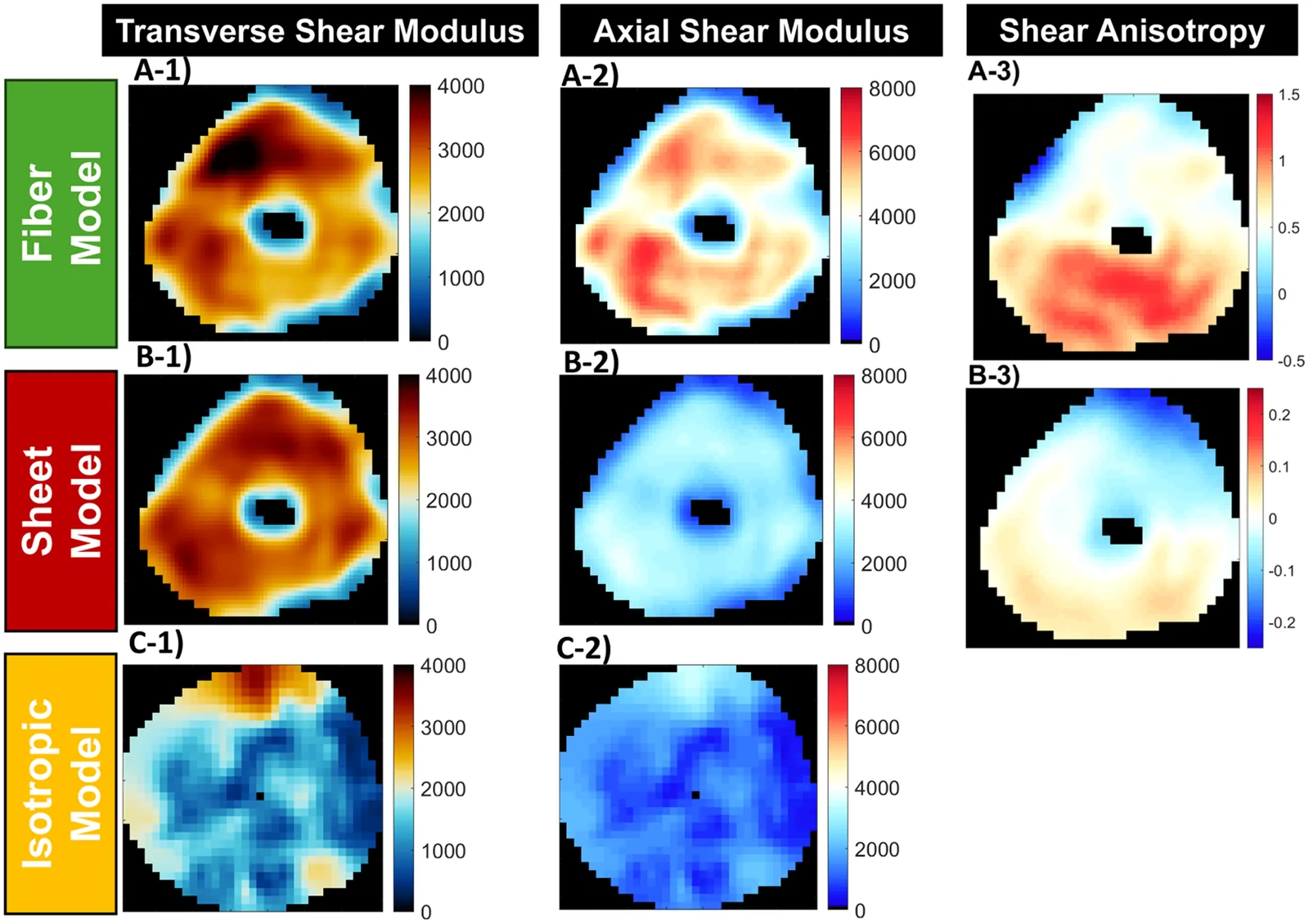
The fiber model (A), sheet model (B), and isotropic incompressible model (C) show mappings of (1) the transverse shear modulus $\left(\mu_{\text{tra}}\right)$, (2) the axial shear modulus $\left(\mu_{\text{ax}}\right)$, (3) and the shear anisotropy (ϕ). In the isotropic model, the transverse and axial shear moduli are identical (ϕ=0). Results are from the second slice of Specimen #1, left ventricle, acquired at the basal plane.

**Fig. 6.**
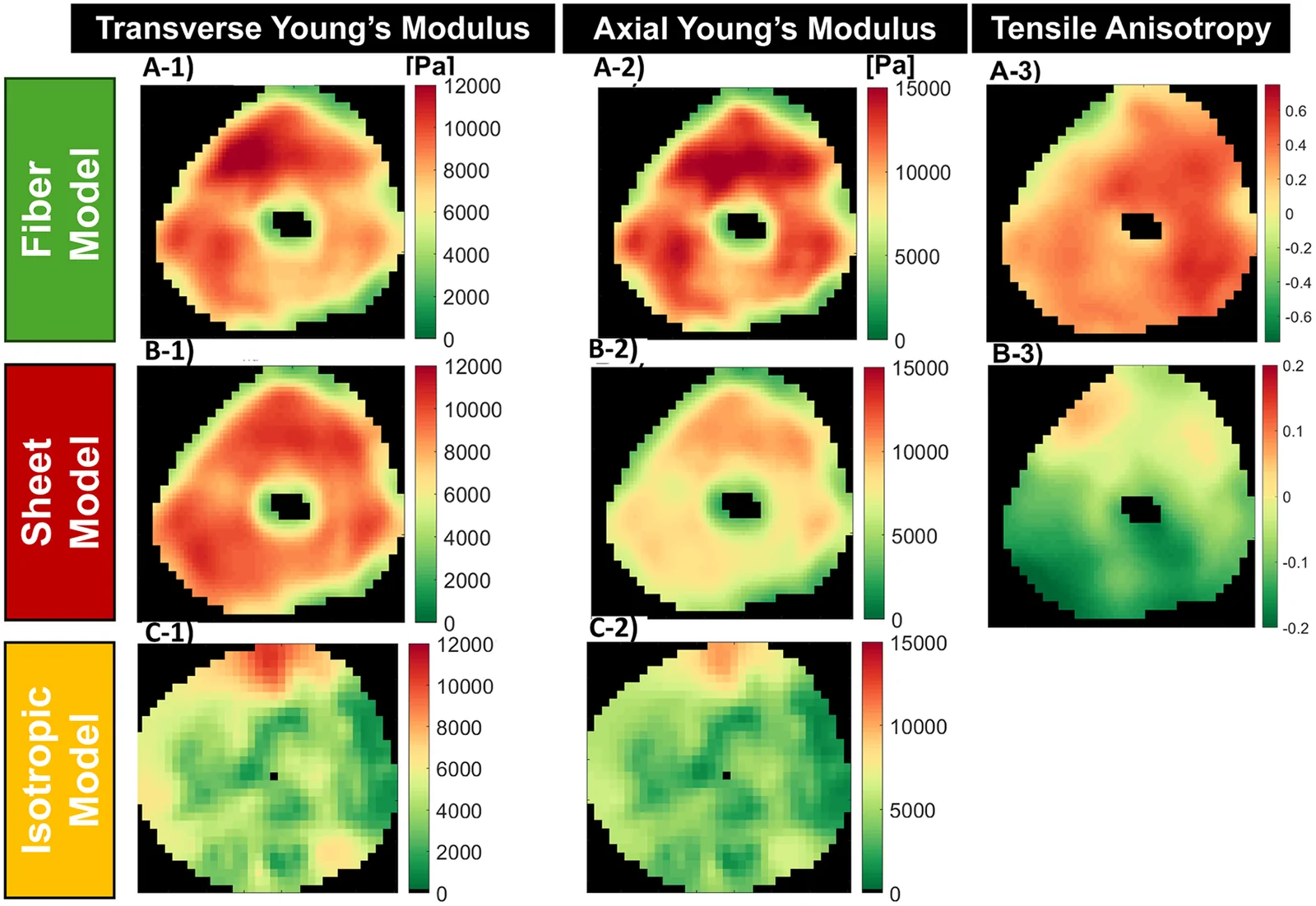
The fiber model (A), sheet model (B) and isotropic incompressible model (C) show mappings of the Young’s modulus (E) in either (1) transverse $\left(E_{\text{tra}}\right)$ or (2) axial $\left(E_{\text{ax}}\right)$ orientations, along with the tensile anisotropy (ζ). Note that in the isotropic model, the transverse and axial Young’s moduli are identical (C-1 is identical to C-2, ζ=0). Results are from the second slice of (Specimen #1, left ventricle). The second slice, acquired at the basal plane, is shown.

We were able to obtain converged mechanical property distributions for the fiber, sheet and isotropic model solutions via TI-NLI and standard NLI MRE reconstruction, enabling analysis across the myocardial wall. Figs. 5 and 6 show the heterogeneous mechanical property distribution within the myocardium.

**Fig. 7.**
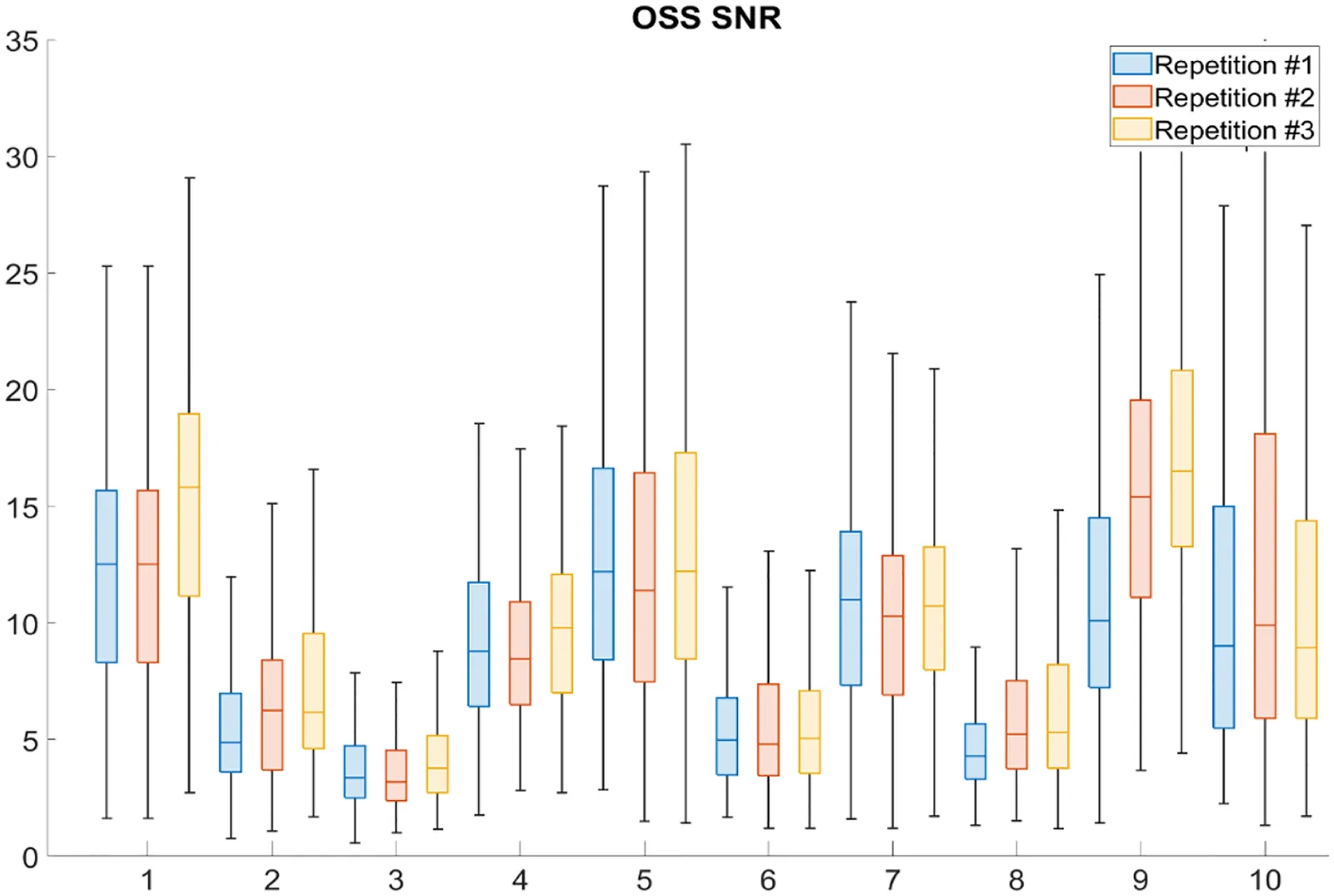
Repeated measures of the OSS SNR at each new MRE acquisition in each specimen in the LV. The first quartiles are the boxes and the whiskers represent the interquartile range.

The average OSS-SNR value across specimens and repetitions was 9.25±6.01 in the LV (Fig. 7).

Convergence of MRE metrics was reached for all experiments with displacements errors below 20 % in both amplitude and phase (Figure S.2, S.3, S.4, S.5, and S.6).

### 3.2. Variation across the myocardial wall

The isotropic model does not distinguish between axial and transverse components $\left(E_{\text{ax}}=E_{\text{tra}}=3\mu_{\text{tra}}=3\mu_{\text{ax}}\right)$. Consequently, some specimens showed a lack of physiological patterns in the mapping of shear and Young’s modulus distributions (Fig. 5 and Fig. 6, *e.g*., specimen #1). Overall, MRE metrics from the isotropic model were of the same order of magnitude as those from the anisotropic model but exhibited systematic biases: a downward bias at mid-wall, an upward bias at the epicardium, and an inability to distinguish specific cardiac deformation modes. The isotropic model estimated stiffness values 24 % to 31 % lower than those obtained with the two anisotropic models (Table 1).

In the fiber model, the shear modulus for the (FN, FS) shear deformation modes reached $\mu_{\text{ax}}=1.9\pm 0.1\;\text{kPa}$ at mid-wall and ~ 0.7 ± 0.03 *kPa* at the peripheries while in the sheet model the shear modulus for the (SN, NF) shear deformation modes reached 1.5 ± 0.1 *kPa* at mid-wall and 0.9 ± 0.04 *kPa* at the peripheries, showing good agreement on the stiffness of this shear deformation modes between the two anisotropic models. Overall, both anisotropic models found fibers at the mid-wall to be roughly twice as stiff in shearing as those in the endocardium or epicardium. Similar findings were observed in the Young’s moduli (Table 1).

#### 3.2.1. Shear modulus

In the fiber model, there was a significant difference between shearing along the fiber axis, $\mu_{\text{ax}}=(\text{FN},\;\text{FS})\;=\;1956\pm 140\;Pa$, at the mid-wall compared to the outer layers (962 ± 59 *Pa* in the endocardium and 936 ± 56 *Pa* in the epicardium, P<0.0001), equivalent to a 203 % increase in stiffness within the mid-wall (Table 1).

Shearing in the plane normal to the fiber axis at mid-wall, $\mu_{\text{tra}}=\left(\text{SN},\;NS\right)=1277\pm 107\;Pa$, was 172 % stiffer than in the outer layers (714 ± 28 *Pa* in the endocardium and 786 ± 102 *Pa* in the epicardium, P<0.0001).

As a result, sheetlets sliding on each other at mid-wall along the fiber axis, $\mu_{\text{ax}}$, experienced greater shearing resistance than sheetlets sliding against the fiber axis (*i.e.* $\mu_{\text{tra}}$ perpendicular to the fiber axis) ($\mu_{\text{tra}}=65\%\;\mu_{\text{ax}},P<0.0001$ between $\mu_{\text{tra}}$ and $\mu_{\text{ax}}$). The resistance to a specific shearing direction ($\mu_{\text{ax}}\;vs\;\mu_{\text{tra}}$) remained significant even in the endocardium $\left(\mu_{\text{tra}}\;=\;74\%\;\mu_{\text{ax}},\;P\;<\;0.0001\right)$, and in the epicardium $\left(\mu_{\text{tra}}\;=\;83\%\;\mu_{\text{ax}},\;P\;<\;0.0007\right)$ (Table 1), but to a lesser magnitude.

In the sheet model, which did not include specific deformation modes corresponding to shear along or against the fiber axis, there were no significant differences at mid-wall between $\mu_{\text{tra}}$ and $\mu_{\text{ax}}(\mu_{\text{tra}}\;\approx \;\mu_{\text{ax}},\;P\;=\;0.8984)$. Similarly, the resistance to a specific shearing direction $\left(\mu_{\text{ax}}\;vs\;\mu_{\text{tra}}\right)$ remained significant in the endocardium $\left(\mu_{\text{tra}}=109\%\;\mu_{\text{ax}},P=0.0004\right)$, but not in the epicardium $\left(\mu_{\text{tra}}=103\%{\;\mu }_{\text{ax}},P=0.0726\right)$ (Table 1).

#### 3.2.2. Young’s modulus

In the fiber model, stretch resistance along the fiber axis $\left(E_{\text{ax}}\right)$ changed throughout the myocardium wall reaching 5631 ± 443 *Pa* at the mid-wall, 2844 ± 314 *Pa* at the endocardium (50.5 %, P<0.0001 with mid-wall value), and 2777 ± 261 *Pa* at the epicardium (49 %, P<0.0001 with mid-wall value). Thus, $\left(\text{FF}\;\text{midwall})=\frac{1}{2}(\text{FF}\;\text{endocardium})=\frac{1}{2}(\text{FF}\;\text{epicardium}\right)$.

For stretching perpendicular to the fiber $\left(E_{\text{tra}}\right)$ also changed throughout the myocardium wall reaching 3832 ± 321 *Pa* at the mid-wall, 2142 ± 85 *Pa* at the endocardium (55.8 %, P<0.0001 with mid-wall value), and 2358 ± 306 *Pa* at the epicardium (61 %, P<0.0001 with mid-wall value).

Stretch resistance along the fiber axis $\left(E_{\text{ax}}=5631\;\pm \;443\;Pa\right)$ predominates compared to stretch resistance perpendicular to the fiber axis $\left(E_{\text{tra}}=3832\;\pm \;321\;Pa\right)$ (P<0.0001). It was also true regardless of transmural position, at the endocardium $\left(E_{\text{ax}}\;=2844\pm 314\;Pa,\;E_{\text{tra}}\;=2142\pm 85\;Pa,\;P<0.0001\right)$, and at the epicardium $\left(E_{\text{ax}}=2777\pm 261\;Pa,\;E_{\text{tra}}=2358\pm 306\;Pa,\;P=0.0040\right)$.

Since the sheet model was derived from a rotation of the constitutive matrix to align with the third eigenvectors of the diffusion tensor, it should exhibit equivalences with the fiber model (Table 1):

At the mid-wall:

$$E_{\text{tra}}(\text{Fiber}\;\text{Model})\approx E_{\text{ax}}(\text{Sheet}\;\text{Model})(P=0.4776),$$

$$E_{\text{ax}}(\text{Fiber}\;\text{Model})\neq E_{\text{tra}}(\text{Sheet}\;\text{Model})(P=0.0001).$$

At the endocardium:

$$E_{\text{tra}}(\text{Fiber}\;\text{Model})\neq E_{\text{ax}}(\text{Sheet}\;\text{Model})(P=0.0137),$$

$$E_{\text{ax}}(\text{Fiber}\;\text{Model})\approx E_{\text{tra}}(\text{Sheet}\;\text{Model})(P=0.8536).$$

At the epicardium:

$$E_{\text{tra}}(\text{Fiber}\;\text{Model})\approx E_{\text{ax}}(\text{Sheet}\;\text{Model})(P=0.0412),$$

$$E_{\text{ax}}(\text{Fiber}\;\text{Model})\approx E_{\text{tra}}(\text{Sheet}\;\text{Model})(P=0.8619).$$

**Fig. 8.**
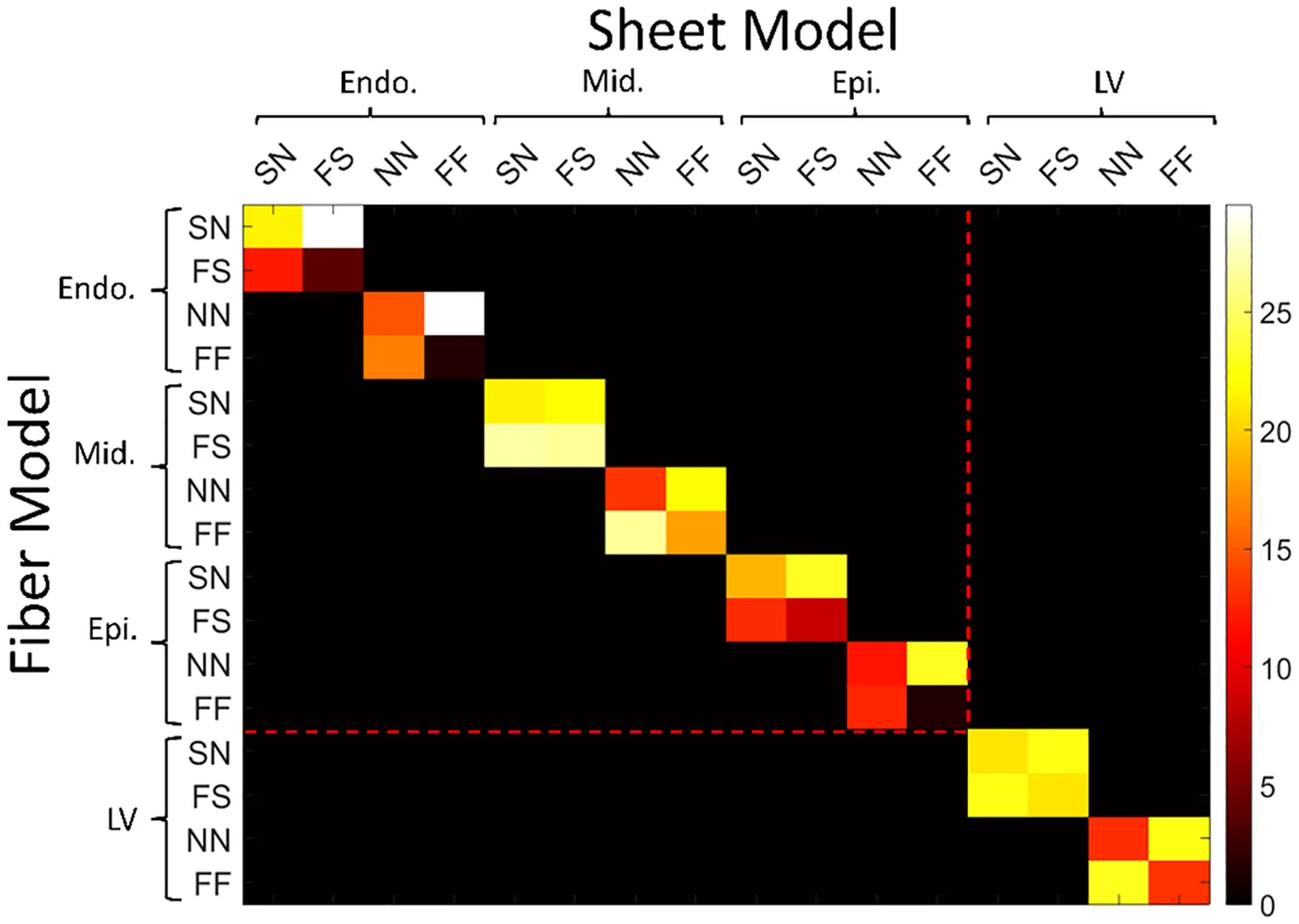
Relative mean absolute differences, in [ %], between the fiber model sheet models, across each mode within the endocardium (endo.), mid-wall (mid.), and epicardium (*epi*.).

In addition, the relative mean absolute difference between deformation modes from the fiber model and the sheet model shows that the deformation modes are relatively consistent regardless of the model selected (matrix cross diagonal in Fig. 8). For instance, the deformation modes retrieved from Young’s modulus, FF and NN mode, showed only 13 % RMD between the two models for the entire LV. SN and FS mode showed only 20 % RMD between the two models for the entire LV.

### 3.3. Comparison with literature

Stiffness metrics for the entire left ventricle are presented for each model and compared with values reported in the literature in Table 2. A more detailed presentation of these reported values is provided in Table S.2.

Two tailed Welch’s statistical t-tests with adjusted P-values (Holm-Bonferroni Method) showed that our aMRE models (fiber and sheet) were not significantly different from each other, and from our isotropic model, given the dispersion of stiffness measurement when considering all ten specimens at once (Figure S.7, all specimens 1:10). There were significant differences across models within the same specimen (Figure S.7).

Using aMRE, shear moduli was found in the range 1.17 ± 0.74 to 1.78 ± 1.34 kPa, which compares well with FE studies, with simulated or acquired fiber field, reporting 1.2 ± 0.4 kPa [47] (P=0.2189 with our reported transverse shear modulus from aMRE at 1.78 ± 1.34 kPa), 1.60 ± 0.22 kPa [49] (P=0.6849), 1.94 ± 0.07 kPa [46] (P=0.7148) in human hearts. Some recent mechanical load test studies in human myocardium showed a similar ranges of 1.28 kPa [42], 1.43 kPa [52], 1.75 kPa [42], 1.42 ± 0.35 kPa (P=0.4302) [53] while older parametric models did not, reporting values from 5.5 ± 2.0 (P<0.0002) to 7.0±3.0kPa(P<0.0001) [52], 5.75 ± 1.5 to 11.5 ± 2.5 kPa [52], and 13 ± 3.25 [60] (P<0.0001). MRE with isotropic models ranged 3 to 5-fold higher (3.0 ± 0.6 [13] (P=0.0220 with aMRE shear moduli at 1.78 ± 1.34 kPa, P<0.0001 with 1.17 ± 0.74 kPa) to 8.9 kPa [7]) in pigs than our aMRE models (Table 2). This difference of stiffness between *ex vivo* and *in vivo* measurements will be further discussed.

On the other hand, Young’s moduli, $E_{\text{ax}}=5.01\pm 3.42\;\text{kPa}$ and $E_{\text{tra}}\;=3.52\pm 2.23\;\text{kPa}$ with the fiber model, had mixed results compared to reported values in FE and mechanical load test studies. There were similarities with some FE studies in pigs, reporting $E_{\text{tra}}=5.57\;\text{kPa}$ [16] or 4.44 kPa [42] and in humans, reporting 2.26 kPa [45, 42], 3.13 kPa [54], 4.27 kPa [56,57], 4.44 kPa [42], 5.48 kPa [55] (Table 2 and Table S.9). However, our reported $E_{\text{ax}}$ was similar to only a few other FE studies, reporting 3.05 kPa [58], 5.88 kPa or 8.46 kPa [50], and 3.04 kPa [42] to 5.0 kPa [52] in mechanical load tests studies.

Overall, equibiaxial extension testing, especially the axial Young’s modulus, was 3 to 30 times higher than our proposed aMRE estimation (Table 2). This difference will be further discussed.

The reported values for FE studies on the axial Young’s modulus tend to be at the higher end of the spectrum, possibly due to the controlled and idealized conditions under which these models operate. The measured values from our study align better with lower reported values, suggesting that *in vivo* and *ex vivo* measurements might capture additional complexities not accounted for in some finite element models. The fact that our aMRE measures were taken in the passive state may also have an influence on these results.

### 3.4. Repeatability of the MRE metrics

Within-subject standard deviation, with the fiber model, shows repeated scans within a single heart vary by ±114.72 Pa from the average axial shear modulus value such that for repeated aMRE measurements of $\mu_{\text{ax}}$, on the same heart under identical conditions, 95 % of the measurements will fall within ±318.0 Pa of each other (Table 3).

Young’s moduli had the largest RC range, on the order of ±0.5 kPa in the sheet model and ±[0.6–0.9] kPa in the fiber model. Shear moduli had a lower RC range, meaning better repeatability, in the sheet model (±0.17 kPa) compared to the fiber model (±[0.2–0.3] kPa). The sheet model and its shear moduli also exhibited the lowest mean bias (3 Pa; 95 % CI:[-143,149]Pa), whereas the Young’s moduli in the fiber model had the highest mean bias (− 197 Pa; 95 % CI:[-975,580]Pa) (Table 3).

**Fig. 9.**
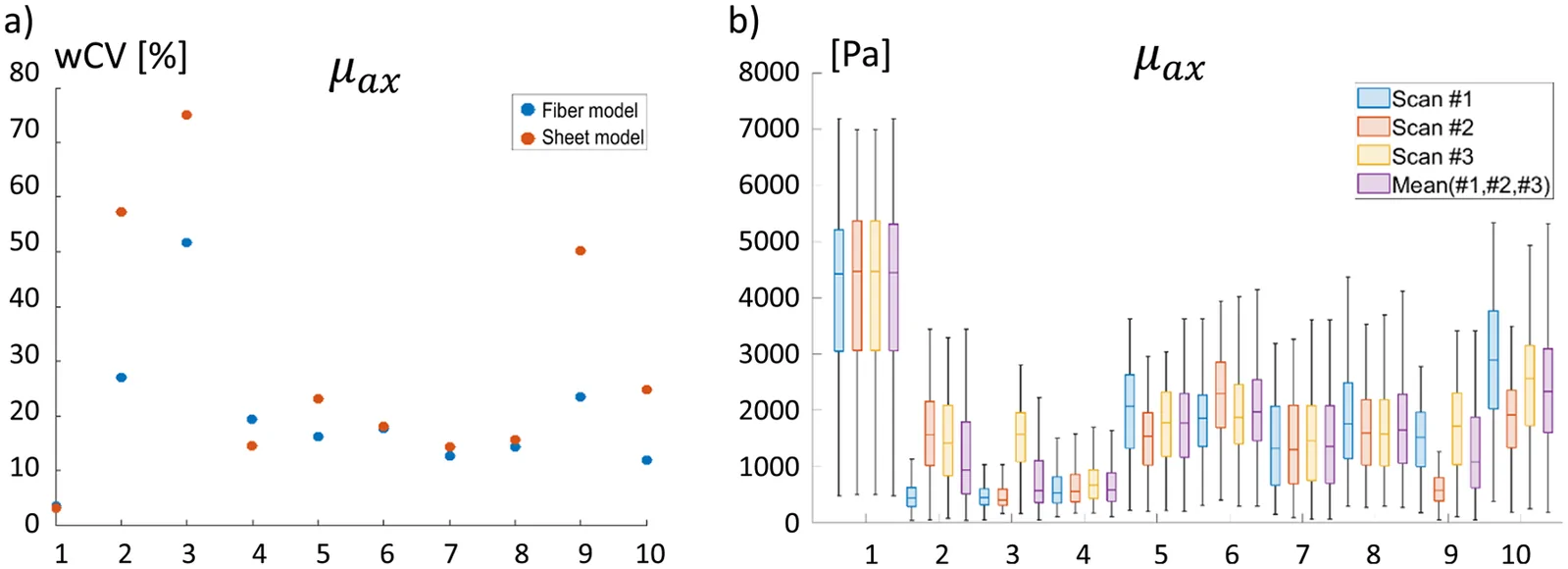
Within-sample coefficient of variation in the ten specimens for each model (3 repeated scans) (a) and their respective boxplot for axial shear modulus (median and [25 %:75 %] percentiles box limit) (b).

Considering values for repeated scan at a single metric, Fig. 9 shows that the wCV for $\mu_{\text{ax}}$, remained within 15 % for seven of the ten specimens.

From Fig. 9-a, higher within-sample wCV (>20 %, specimens #2, #3, and #9) originates from non-repeatable measurements (Fig. 9-b) where the mechanical driver may have been misplaced, leading to lower OSS-SNR (OSS-SNR <5) (Fig. 7). In the isotropic model, an OSS-SNR above three is recommended [40]. Anisotropic MRE may require higher OSS-SNR as more parameters are estimated from the data. Nevertheless, Table 3 and Fig. 9 show that despite occasional manual errors in the setup which were corrected, good agreement was obtained within repeated measures (*i.e*., mean bias within the 95 % LOA, indicating accuracy), along with high precision (*i.e*. small 95 % CI), and acceptable repeatable coefficient (± [0.17–0.9] kPa).

Lin’s concordance correlation coefficient (CCC) confirmed the high scan-wise repeatability of aMRE. Across ten hearts and three acquisitions the CCC for Young’s and shear moduli ranged 0.82–0.92 (Table 3), indicating *substantial to almost-perfect* concordance. Anisotropy indices ϕ and ζ also showed good concordance (0.69–0.91). These values compare favorably with previously reported CCCs for cardiac MRE (0.70–0.88) and ultrasound shear-wave imaging (0.60–0.85) [62–64]. The sheet model yielded slightly higher CCCs than the fiber model, in line with its narrower Bland-Altman limits of agreement.

Variations in global stiffness measurements across the specimens were observed (Figure S.7). When a specimen was stiffer than others in one direction, *e.g*. along the fiber axis, it was also stiffer in other directions, such as in the plane of its sheetlets and normal to its sheetlets.

For the fiber model, the average shear anisotropy was ϕ=0.46±0.39 and the average tensile anisotropy was ζ=0.41±0.46. In contrast, for the sheet model, the corresponding values were ϕ=-0.01±0.15 and ζ=-0.08±0.17, respectively. This difference in effective anisotropy levels is possibly due to the fact that the transverse orientation in the anisotropic sheet model combined effects along and against the local fiber axis. The negative anisotropy levels for the sheet model indicate that the material was stiffer in the transverse orientation than in the axial orientation, which corresponds well with the fact that the transverse orientation is this case combines effects along and against the local fiber axis, where the effects along the fiber axis would lead to an overall increase in stiffness in this transverse orientation.

## 4. Discussion

### 4.1. Comparison of the isotropic, fiber, and sheet models

This study demonstrates how diffusion tensor imaging can enhance MRE by enabling anisotropic MRE, which formalizes *fiber* and *sheet*-based anisotropy models for transversely isotropic materials. With this method, we successfully identified and mapped axial and transverse shear and Young’s moduli in the myocardium, which were further categorized into the following cardiac deformation modes: (SN or NS), (FN or FS), (SF or FS), (SN or NF), (FF), (NN or SS), (NN), and (FF or SS).

Both the *fiber* and *sheet* models improved elastic characterization of the myocardium compared to isotropic MRE [7,9–13,43,44,51], yielding moduli values better aligned with literature-reported Finite Element (FE) studies [16,42,45–47,49,50,54,55,58,59], and some more recent invasive studies [42,53],.

The method proved reliable, as within-subject repeatability of $\mu_{\text{ax}},\;\mu_{\text{tra}},E_{\text{ax}},\;\text{and}\;E_{\text{tra}}$ was achieved in 27 out of 30 repeated measures.

When the analysis was performed across all specimens on the LV, the sheet and fiber models were not significantly different from each other and from our isotropic model. Reported variation across specimens was principally due to outliers (*e.g*. specimen #2 and #3) and natural stiffness variation across specimens. Analysis by single specimen showed significant differences between the anisotropic models and the isotropic model.

The sheet and fiber models were generally in agreement (<10 % relative difference) concerning the stiffness of different shear (SN, FS) and tensile (FF, NN) deformation modes within the myocardium. However, it is important to note that these stiffness values were obtained from *different* anisotropic stiffness components of the two models. For example, the FF mode corresponds to E_ax_ in the fiber model and E_tra_ in the sheet model. Thus, these similarities in the measured stiffness between cardiac modes were obtained thanks to the two models’ ability to account for the anisotropy of the myocardium during characterization.

The anisotropic models estimated myocardial stiffness values 24–31 % higher than those obtained using the isotropic model in these *ex vivo* hearts. This discrepancy is consistent with findings in other anisotropic tissues, such as the brain, where Anderson et al. [18] reported similar limitations of isotropic MRE reconstruction. The TI-NLI algorithm for aMRE reconstruction has been validated in both isotropic and anisotropic MRE phantoms [37], suggesting that this approach might provide more physiologically relevant stiffness estimates.

Nevertheless, previous results *in vivo* cardiac MRE based on isotropic models have shown good interscan agreement (concordance correlation coefficient of 0.77) with methods extensively validated in both heart phantoms and relative to dynamic mechanical testing [62–64]. The end-diastolic stiffness observed *in vivo* isotropic MRE experiments [7,9, 10], generally agree with the aMRE results reported here once the *ex vivo* nature of the tissue samples evaluated in this study is considered.

The isotropic model in this study showed shear modulus values on the order of 1 kPa which corresponds to a shear wavelength of roughly 10 mm, which is not observed in the acquired displacement data. However, this simple consideration of the observed wavelength in the myocardium can be very misleading, as structural wave propagation dominates. Observed wavelengths are significantly longer in structural waves, such as Lamb and Rayleigh waves, than those observed in pure shear weaves within a given material. The subzone NLI method is not a wavelength based elastography algorithm and instead uses a complete model for viscoelastic wave propagation, which accounts for surface reflections and material heterogeneities, and as such can distinguish the underlying stiffness even in the presence of structural wave propagation.

Finally, both anisotropic models indicated that myocardial elasticity exhibits directional dependence, aligning well with myofiber microstructure. These findings highlight the importance of differentiating between axial and transversal shear and tensile elasticity for a more accurate characterization of myocardial stiffness.

### 4.2. Repeatability of aMRE metrics

Across all Bland-Altman analyses, the line of equality remained within the 95 % confidence interval (CI), confirming high within-subject repeatability for $\mu_{\text{ax}},\mu_{\text{tra}},E_{\text{ax}},\;\text{and}\;E_{\text{tra}}$.

The sheet model exhibited lower mean bias and within-subject variability than the fiber model. This difference may arise from the reduced impact of the precise orientation of the axis of material symmetry for this model, where the local fiber orientation was combined with a cross-fiber orientation and therefore had a mixed impact on overall stiffness.

The repeatability coefficient (RC) was within a range within ±0.2 kPa for the shear moduli and within ±[0.5–0.9] kPa for the Young’s moduli. In addition, Young’s moduli exhibited greater mean bias and wider confidence intervals than the shear moduli. Notably, in specimens #2 and #3, low OSS-SNR during the first scan resulted in underestimated stiffness values. However, correcting the passive driver placement in these cases increased estimated stiffness values by a factor of 4–5, suggesting that the recommended OSS-SNR threshold of 3 [40] may not be suitable for static *ex vivo* cardiac aMRE.

### 4.3. Young’s modulus: anisotropic model values and correspondence with cardiac deformation modes

Stretch resistances along the fiber axis were highest at mid-wall (roughly twice as stiff), and, to a lesser extent, at the epicardium, compared to stretching perpendicular to the fiber axis. In the fiber model, the axial Young’s modulus (aligned with the primary fiber direction, *e.g*. the FF tensile deformation mode), was distributed through myocardium as:

$$E_{\text{ax}}=5.63\pm 0.44\;\text{kPa}\left(\text{LV}\;\text{midwall}\right),$$

$$E_{\text{ax}}=2.84\pm 0.31\;\text{kPa}(\text{epicardium},\;P<0.00001)$$

and

$$E_{\text{ax}}=2.77\pm 0.26\;\text{kPa}(\text{endocardium},\;P<0.00001).$$

Given the changes in fiber orientations through the myocardium, these results indicate that, in the passive case, circumferential fibers at mid-wall require twice the force to achieve the same deformation as longitudinal fibers at the epicardium or endocardium.

Anisotropic Young’s moduli, $E_{\text{ax}}$ for FF tensile deformation mode and $E_{\text{tra}}$ for the NN or SS deformation modes, were more aligned with FE studies and some mechanical load tests which accounted for fiber orientation, compared to those obtained from the isotropic MRE model. Previous *in vitro* measurement of myocardial elastic moduli demonstrated nonlinear anisotropic mechanical behavior, with greater stiffness along the fiber direction compared to the cross-fiber direction. These findings are consistent with our results [16,52,61,65–67]

### 4.4. Shear modulus: anisotropic model values and correspondence with cardiac deformation modes

Shear resistances along the fiber axis, $\mu_{\text{ax}}=(\text{FN},\;\text{FS})$, were also significantly stiffer at mid-wall than the outer layers (endocardium and epicardium). Additionally, regardless of the myocardial layer, these sheetlets sliding on each other along the fiber axis showed significantly greater shearing resistance than sheetlets sliding against the fiber axis, $\mu_{\text{tra}}=(\text{SN},\;NS)$. Sommer et al. reported the following hierarchy of stiffness [52]: F-direction (fiber - aligned) > S-direction (sheet-aligned) > N-direction (normal to sheet).

The FN and FS modes, which represent shearing along the primary fiber axis, exhibited the highest stiffness. In contrast, the SF and SN modes had intermediate stiffness properties, whereas the NF and NS modes had the lowest stiffness [1]. These observations are consistent with our results.

## 5. Implications

### 5.1. Stiffness and myocardial architecture

The human myocardium is less stiff than porcine myocardium, a critical factor in cross-species comparisons. This difference is likely due to variations in fiber angulation across species [52,68]. Although it can be cost effective to study rodent hearts, they have a markedly different structural organization [68]. The increased fibre angle range results in increased Cauchy stress values for each mode [69]. As a result, rodent myocardial stiffness studies were excluded from our comparison (Table 2, and Table S.2 to Table S.12).

FE studies show that altering fiber and sheetlet angles directly influences myocardial stiffness values [48,50,55,57,70]. For example, Li *et al*. compared helical fibers centered at 0° at mid-wall *vs*. helical fibers linearly increasing from 0° to 90° They observed a 2.5-fold increase in $E_{\text{ax}}$ and $\mu_{\text{tra}}$, and a 3–6 fold decrease in $E_{\text{tra}}$ and $\mu_{\text{ax}}$, respectively [48]. Most FE studies simulate fiber angles with a range of [− 60° : + 60°] for the human heart (Table S.2 to Table S.8). However, X-ray phase contrast measurements ($3.5\;{\mu m}^{3}$ resolution) showed that fiber angles are segment-dependent, with a range of [− 90° : + 100°] [71,72]. This discrepancy may introduce errors in the stiffness estimations from FE studies.

Myocyte helix angulation increases from diastole to systole [73], potentially correlating with stiffness increases observed *in vivo*. Probing myocyte angulation changes may inform indirectly on the heart stiffness and thus represent a marker for myocardial performance and health. Patients with tetralogy of Fallot, transposition of the great arteries, and single ventricles exhibit reduced fiber angulation in specific myocardial segments [26]. These abnormalities may alter myofiber stress distributions at end-diastole, affecting stiffness and cardiac function [50].

### 5.2. What is the ground truth in stiffness measurements?

Stiffness is inherently influenced by myocardial architecture. Our data indicates greater stiffness in the fiber direction, aligning with findings from FE studies and some more recent mechanical load test studies using the general Holzapfel and Ogden model [42,53], while older studies not using this model did not agree [52,60]. These more recent studies assumed a linear helix centered at mid wall (0°) with fiber angle in the range [− 96°: +96°], which is similar to our measurements.

This suggests that accounting for fiber angulation is crucial for obtaining repeatable stiffness measurements, as it reflects the state of the myocardial architecture during measurement.

As techniques using ultrasound shear wave velocity, acoustic radiation force impulse, and pressure-volume loops disregard tissue architecture, these cannot serve a comparison with aMRE. Instead, we concentrated on studies involving swine, sheep, and human hearts, utilizing MRE, FEM, and mechanical load tests, where muscle fiber angulation is known. Additionally, FE studies often incorporate DTI data in their modeling. By focusing on these specific methodologies, we ensure a meaningful comparison across similar elastography methods and heart shapes.

Mechanical load tests generally report higher stiffness values, as they can apply controlled loads that may not be physiologically representative but are useful for understanding the maximum capacity of the tissue. The aMRE stiffness results presented here are generally lower compared to these mechanical load tests results, which is expected given the non-destructive, small deformation nature of MRE measurements. Mechanical load tests apply high levels of strain to the passive tissue, up to the point of rupture. These deformation levels are in a different elastic regime compared to the small deformations often used in MRE and FE studies. For example, our aMRE approach uses a linear elastic, infinitesimal strain model with no geometrical nonlinearity. The TI-NLI algorithm for aMRE reconstruction has been validated in both isotropic and anisotropic MRE phantoms [37], demonstrating that this approach is able to provide physiologically relevant stiffness estimates. Overall, both methods, aMRE and mechanical load tests, can provide true measurement of stiffnesses in their respective elastic regime, but care must be taken when comparing them. In addition, stiffness measurements should always be accompanied by fiber angulation data, regardless of the methodology (*e.g*., aMRE, FEM, mechanical loading tests). Techniques that estimate myocardial stiffness without accounting for fiber angulation may be unreliable [6]. Since our repeatable aMRE method aligns with literature values, we anticipate similar agreement in eventual human studies.

### 5.3. Toward in vivo aMRE applications

This study used low-amplitude external vibrations to induce small-strain displacement fields, which were measured with MRI and analyzed via aMRE reconstruction. These small strains align with linear elasticity assumptions, which have been widely validated in phantom and *in vivo* studies [74–77].

For successful *in vivo* applications, the cardiac aMRE procedure would require:

1. Low-amplitude vibrations synchronized with cardiac-gated MR imaging,
2. Real-time analysis of instantaneous myocardial stiffness throughout the cardiac cycle,
3. Integration of these instantaneous stiffness measurements into nonlinear models of the myocardial wall mechanics.

Current research is focused on refining *in vivo* aMRE methods to enable non-invasive myocardial stiffness assessment. Future studies should aim to (1) optimize MRE acquisition parameters for clinical feasibility, (2) refine computational models, *e.g*. using a two-fiber model [78], to account for non-linear myocardial behavior.

Despite its current limitations, aMRE offers a promising pathway for non-invasive myocardial characterization, bridging the gap between MR imaging and mechanical property assessment.

## 6. Study limitations

### 6.1. Limitations of the TI-NLI model

The fiber model exhibited greater axial variability across specimens than the sheet model (with twice the interquartile range). This variability may be attributed to challenges in determining the primary eigenvector when OSS-SNR in MRE is low or when DTI SNR is insufficient.

By design, the TI-NLI aMRE framework isolates a single spatial axis to differentiate axial and transverse elasticity. However, a single-axis model cannot fully capture the myocardial microstructural complexity. The myocardium exhibits regional structural variations, including differences in size, thickness, number and orientation of the sheetlets and cleavage planes [14,71,72]. These microstructures affect collagen distribution which influences elasticity and shear properties.

Moreover, there is evidence of two orthogonal populations of sheetlets (along n1 and s1) [14,15]. Our study only considered a single sheetlet population along n1. With aMRE, the tensile deformation modes FF and NN can be determined by the fiber and sheet models, respectively, corresponding to the Young’s modulus for the fibers and the first orthogonal sheetlet population. Applying the TI axis of axis of symmetry to s1 would result in obtaining the following deformation mode combinations $\mu_{\text{ax}}=(\text{SN}\;\text{or}\;\text{FS}),\;\mu_{\text{tra}}=(\text{FN}\;\text{or}\;\text{NF}),\;E_{\text{ax}}=(\text{SS}),\;\text{and}\;E_{\text{tra}}\;=(\text{FF}\;\text{or}\;\text{NN})$, which can help to isolate other deformation modes than those presented in this study. Nevertheless, a two-fiber model would provide a more accurate representation of myocardial mechanics [78], but its complexity extends beyond the scope of this study.

### 6.2. Approximations in the fiber and sheet models

Both fiber and sheet models have their approximations.

In the fiber model, we assume one dominant fiber orientation, treating all other directions as “perpendicular” to the fiber, thereby ignoring the sheetlet structure.

In the sheet model, we assume that all directions within the sheet plane exhibit identical mechanical properties, which is not necessarily valid if the fibers within the sheet are strongly anisotropic.

Additionally, neither model accounts for intersecting sheet orientations, despite evidence of orthogonal sheetlets [14]. Two-fiber models for aMRE do exist and should be further investigated in future studies [78].

Despite these limitations, aMRE reconstruction – which combines tensile and shear deformation mode assessments – aligned well with FE studies and some mechanical loading studies. This level of agreement was not observed in previous cardiac MRE studies, which predominantly focused solely on the mean values of the transverse shear modulus $\left(\mu_{\text{tra}}\right)$ using isotropic models.

### 6.3. Limitations of freshly excised heart specimens

It is important to note that these previous porcine cardiac MRE results (Table 2) were obtained *in vivo*, compared to the *ex vivo* results presented here. This has a two-fold impact on the estimated stiffness from MRE. Firstly, an *in vivo* heart has residual internal pressure that could modify the effective stiffness of the tissue detected by MRE. Previous studies have measured the end-diastolic pressure in porcine hearts at slightly over 10 mmHg (1.33 kPa) [7,9,10]. Secondly, *in vivo* tissues are perfused and stimulated, which can modify the effective stiffness when compared to even recently harvested *ex vivo* tissues. An MRE-based study of porcine brain tissue found *in vivo* tissue to be roughly 30 % stiffer than *ex vivo* tissue at 100 Hz [79]. The cumulative effect of these two aspects could explain the stiffness differences observed between previous *in vivo* MRE studies and the *ex vivo* results reported here.

Our findings provide insights into myocardial stiffness in freshly excised hearts under a specific contraction state. However, the study’s reliance on *ex vivo* specimens introduces certain limitations. While pentobarbital euthanasia is widely used in cardiac studies and is generally believed to preserve myocardial tissue effectively, subtle stiffness changes may still occur due to the procedure itself.

To minimize postmortem alterations, it was crucial to handle and scan the hearts within one hour after euthanasia. Delayed scanning could lead to rigor mortis, which typically begins around two hours postmortem and results in progressive tissue stiffening. This phenomenon could potentially alter the mechanical properties of the myocardium and affect the accuracy of stiffness measurements. Despite rapid post-euthanasia imaging, *ex vivo* conditions may still introduce deviations from physiological states.

Nevertheless, in clinical and surgical settings, such as cardiac transplantation or intraoperative assessments, the myocardium is often exposed to non-physiological conditions, which may impact its mechanical properties. Under these circumstances, post-surgical aMRE mapping could be valuable for evaluating whether myocardial mechanics are preserved and assessing microstructural factors influencing function.

Additionally, it is important to recognize that myocardial stiffness in *ex vivo* conditions may not fully reflect *in vivo* properties, particularly given that passive myocardial stiffness is influenced by factors beyond fiber alignment, such as extracellular fluid interactions. These viscoelastic properties contribute to stress relaxation behavior, which has been well documented in studies using biaxial extension and triaxial shear testing [58].

Previous studies have also demonstrated that basal shear deformation within the myocardium follows a specific pattern, with shear stiffness decreasing in the order of FS > FN > SN [16]. This pattern suggests that myocardial stiffness is directionally dependent, with certain shear deformation modes exhibiting greater resistance than others. They found that the (FN, FS) deformation modes had the highest stiffness, (SF, SN) deformation modes intermediate stiffness and (NS, NF) deformation modes had the lowest. Our findings align with these observations, supporting the validity of aMRE in capturing myocardial stiffness properties. However, the absence of physiological perfusion and active myocardial contraction in *ex vivo* hearts remain an unavoidable limitation when extrapolating these findings to living cardiac tissue.

## 7. Conclusion

Anisotropic MR elastography (aMRE) is a repeatable and reliable method for measuring axial and transverse shear and Young’s moduli in *ex vivo* swine hearts. By aligning the elasticity matrix with the myocardial microstructure, whether using the fiber or sheet model, aMRE enables the identification of shear and tensile deformation modes that align with previously established myocardial mechanical properties and finite element simulations. These findings confirm that anisotropic elasticity plays a fundamental role in myocardial mechanics, underscoring the importance of accounting for fiber and sheetlet orientations in stiffness characterization.

Compared to isotropic MRE, which systematically underestimates myocardial stiffness, aMRE provides a more physiologically accurate assessment of myocardial mechanical properties. The method successfully captured regional variations in myocardial stiffness, with higher stiffness observed along the fiber axis at mid-wall, consistent with previous mechanical loading studies and computational models.

These advances bring myocardial stiffness assessment closer to clinical application, providing a foundation for a non-invasive methodology capable of true mechanical characterization of the cardiac wall using MR imaging. As aMRE technology continues to evolve, its integration into *in vivo* cardiac assessments holds promise for improving diagnostic accuracy, guiding treatment strategies, and enhancing our understanding of myocardial mechanics in health and disease.

## Supplementary Material

1

Supplementary material associated with this article can be found, in the online version, at doi:10.1016/j.actbio.2025.06.031.

## Figures and Tables

**Table 1:** Shear and tensile moduli in [Pa], calculated as the mean ± standard deviation across repeated measures (n=3), from each median across all the specimens (n=10). The endocardial wall corresponds to the first 10 % of wall thickness, the epicardial wall to the last 10 %, and mid-wall is defined as ±2% depth relative to the zero slope of the second order polynomial fit of the fiber tract across the myocardium. Note that the TI-NLI algorithm considers antagonistic shearing directions to be equivalent, such that SF = FS, NS = SN, and NF = FN.

Segments	Shear & tensile	Fiber model [Pa]		Sheet model [Pa]		Isotropic model [Pa]	
Endocardium	$\mu_{\text{tra}}$	SN or NS	714 ± 28	SF or FS	941 ± 41	FN = FS = SN=SF = NF= NS	993
	$\mu_{\text{ax}}$	FN or FS	962 ± 59	SN or NF	859 ± 44		
	$E_{\text{tra}}$	NN or SS	2142 ± 85	FF or SS	2824 ± 125	FF = NN = SS	2899
	$E_{\text{ax}}$	FF	2844 ± 314	NN	2274 ± 127		
Mid-wall	$\mu_{\text{tra}}$	SN or NS	1277 ± 107	SF or FS	1499 ± 97	FN = FS = SN= SF = NF= NS	807
	$\mu_{\text{ax}}$	FN or FS	1956 ± 140	SN or NF	1505 ± 110		
	$E_{\text{tra}}$	NN or SS	3832 ± 321	FF or SS	4497 ± 291	FF = NN = SS	2422
	$E_{\text{ax}}$	FF	5631 ± 443	NN	3944 ± 368		
Epicardium	$\mu_{\text{tra}}$	SN or NS	786 ± 102	SF or FS	931 ± 39	FN = FS = SN= SF = NF= NS	1067
	$\mu_{\text{ax}}$	FN or FS	936 ± 56	SN or NF	899 ± 36		
	$E_{\text{tra}}$	NN or SS	2358 ± 306	FF or SS	2793 ± 119	FF = NN = SS	3202
	$E_{\text{ax}}$	FF	2777 ± 261	NN	2599 ± 163		

**Table 2:** Measured values across all ten specimens and across the entire left ventricle for the fiber model (FM), sheet model (SM) and isotropic model (IM) are compared with those reported in the literature, expressed in kPa. The types of hearts analyzed include pig (P), human (H), and sheep (S, specifically the right ventricle).

	FM	SM	IM		Literature Comparisons		
				TYPE	FE	MRE (no DTI)	Mechanical load test
$\mu_{\text{tra}}$	1.17 ± 0.74	1.37 ± 0.72	0.97 ± 0.46	P	0.26[42],	3.91±0.39 [43], 3.0± 0.6 [13], 4.94±0.5 [10], 5.56 ±0.39 [9], 8.9 [7], 6.03 ±1.8 [44]	7.20 ± 0.8 [45]
	p-values: FM vs SM: 0.5482 FM vs IM: 0.9580 SM vs IM: 0.4681			H	1.94 ± 0.07 [46], 1.2±0.4 [47], 0.42 [45][42], 0.26 [42], 0.54 [48], 1.60±0.22[49], 0.72 or 1.22 [50]	4.99±1.05 [12], 8.2 [11], 5.6 [51]	1.28 [42], 5.5±2.0 to 7.0±3.0 [52],
				S			1.77±0.71 to 2.13±0.71[53]
$\mu_{\text{ax}}$	1.78 ± 1.34	1.36 ± 0.74		P	0.19[16], 0.26 [42]	2.52±0.31 or 5.53±0.31 [9],	4.20 ± 1.0 [45],
	p-values: FM vs SM: 0.4002 FM vs IM: 0.2940 SM vs IM: 0.3546			H	1.42 [54], 0.60 [55], 0.69 [56][57], 0.15 [58], 2.53 ± 0.05 [59], 2.52 ± 0.05[46], 2.13 [45], 1.08 [45] [42], 0.26 [42], 0.50 [48]		11.6 ± 3.4 [52], 1.43 [52], 1.75 [42], 5.75±1.5 to 11.5±2.5 [52], 13±3.25[60],
				S			1.42±0.35 to 3.55±1.06 [53]
$E_{\text{tra}}$	3.52 ± 2.23	4.13 ± 2.17	2.92 ± 1.37	P	5.57[16], 4.44[42]		11.68 ± 0.8 to 11.9±0.8[45],
	p-values: FM vs SM: 0.4698 FM vs IM: 0.9608 SM vs IM: 0.5435			H	3.13 [54], 5.48 [55], 4.27 [56][57], 1.25 [58], 6.53 ± 0.05 [59], 6.59 ± 0.24 [46], 2.26 [45][42], 4.44 [42], 0.06 [48], 0.88[49][61], 0.01 or 0.34 [50]		47 ± 14 [52], 16.3 ± 6.1 [60], 0.13 [42],
				S			3.19±1.06 to 7.45±3.19 [53],
$E_{\text{ax}}$	5.01 ± 3.42	3.81 ± 2.08		P			13.94 ± 1.8 [45],
	p-values: FM vs SM: 0.5524 FM vs IM: 0.3592 SM vs IM: 0.3009			H	16.18 [54], 29.49 [55], 30.87 [56][57]. 3.05 [58], 11.03 ± 0.63 [59], 10.80 ± 0.48 [46], 8.11 [45], 7.28 [45] [42], 26.77[42], 9.20 [48], 1.55 [49][61], 5.88 or 8.46 [50]		97 ± 32 [52] to 140 ± 56 [60], 5.0 [52], 3.04 [42], 45±6[60],
				S			19.15±5.32 [53]

**Table 3:** Values of: within subject coefficient of variation (wCV), 95 % limits of agreement (LOA), mean bias, 95 % confidence interval (CI), within-subject standard deviation (wSD), repeatability coefficient (RC), and Lin’s concordance correlation coefficient (CCC) for each model and metric, across repeated measures and specimens (pairwise scan comparison).

Model		wCV [ %]	LOA [Pa]	Mean Bias [Pa]	CI [Pa]	wSD [Pa]	RC [Pa]	CCC
Fiber Model	$\mu_{\text{ax}}$	29	[−236, 82]	−77	[−342, 188]	114.72	318	0.86, 0.88, 0.89
	$\mu_{\text{tra}}$	27	[−155, 55]	−50	[−227, 127]	75.76	210	0.83, 0.83, 0.85
	$E_{\text{ax}}$	30	[−661, 266]	−197	[−975, 580]	334.43	927	0.85, 0.84, 0.86
	$E_{\text{tra}}$	27	[−467, 167]	−150	[−683, 382]	228.72	634	0.83, 0.83, 0.85
Sheet Model	$\mu_{\text{ax}}$	19	[−93, 86]	−3	[−153, 147]	64.57	179	0.87, 0.88, 0.89
	$\mu_{\text{tra}}$	19	([84, 90]	3	[−143, 149]	62.77	174	0.88, 0.91, 0.86
	$E_{\text{ax}}$	21	[−293, 243])	−25	[−475, 424]	193.37	536	0.89, 0.87, 0.83
	$E_{\text{tra}}$	19	[−253, 271]	9	[−431, 448]	189.04	524	0.88, 0.91, 0.86
