# Supplementary material for: Distinguishing shear and tensile myocardial wall stiffness using ex vivo anisotropic Magnetic Resonance Elastography
Source: Acta Biomater. Author manuscript; Available in PMC 2026 Aug 28. (PMC13523033; doi:10.1016/j.actbio.2025.06.031)
Supplement: Supplementary file 1 [file NIHMS2183377-supplement-1.docx]

# Supplementary materials

Contents

[1.	Methods	2](#_Toc192444924)

[1.1.	Preprocessing steps	2](#_Toc192444925)

[1.2.	FE model configurations	2](#_Toc192444926)

[1.3.	Convergence criteria for the TI-NLI algorithm.	3](#_Toc192444927)

[2.	Results	4](#_Toc192444928)

[2.1.	Damping ratio	4](#_Toc192444929)

[2.2.	Wave displacement and error	6](#_Toc192444930)

[2.3.	Convergence	7](#_Toc192444931)

[2.4.	Variations Between Samples	9](#_Toc192444932)

[3.	Reported myocardial stiffness in the literature	11](#_Toc192444933)

[Summary	26](#_Toc192444934)

[Bibliography	30](#_Toc192444935)

# 1. Methods

## 1.1. Preprocessing steps

In MRE, motion-encoding gradients are applied during the MRI sequence to encode tissue displacement due to harmonic mechanical vibrations into the phase of the MR signal.

The measured **complex MR signal** $S=Me^{i\Phi}$, where M is the magnitude, Φ is the accumulated phase over the encoding time T such as $\Phi=\int_{0}^{T} \vec{G}(t)\cdot\vec{u}(t)$, the dot product between motion-encoding gradients (G) and tissue displacement with $\vec{u}\left( t \right)=\vec{u_{0}}\left( t \right)cos(\omega t+\Phi)$.

Displacement-induced phase shift images were acquired twice in separate measurements using positive and negative motion encoding gradients, respectively, and subtracted to remove unwanted static background. Phase-difference images were then unwrapped using PhaseTools [1], and Fourier-transformed along the time dimension to extract the harmonic corresponding to the actuation frequency. To reconstruct the temporal waveform**,** the displacement motion can quantified over time from a single complex harmonic as $\vec{u}\left( t_{n} \right)=Re\left( U \right)*cos\left( 2\pi n/N \right)- Im\left( U \right)*sin\left( 2\pi*n/N \right)$, where n=0,1,2,3 and N=4 (number of phase offsets). This gives the estimated displacement at each time point from the complex motion U which can be expressed in polar (A, P) or complex (Ur, Ui) components, such as: $A=abs\left( U \right); P=angle\left( U \right);Ur=A*cos\left( P \right);\mathrm{and} Ui=A*sin\left( P \right).$

The octahedral shear strain was calculated from the complex-valued MRE displacements to ensure that a sufficient measurement quality for the TI-NLI reconstruction was achieved. DTI data were used to align the coordinate system of the transversely isotropic model with the first and third eigenvectors of the diffusion tensor, corresponding to the fiber and sheet model, respectively. No further processing of the MRI and MRE data was performed.

## 1.2. FE model configurations

The FE displacement and property meshes were defined separately following McGarry *et al’* work on multiresolution MRE using nonlinear inversion.

The FE displacement mesh consisted in twenty-seven-node quadratic hexahedral elements fixed to the resolution of the measured MRE displacements (2-mm isotropic voxels) throughout, ensuring an adequate discretization of the displacement field relative to the observed shear wavelength.

The heterogeneous mechanical properties were supported by eight-node linear hexahedral elements of varying resolution across the iterative reconstruction (5-mm isotropic from iteration 1 to 10, 2-mm isotropic from iteration 10 to 70, and 1-mm isotropic from iteration 70 to 300). Refining the resolution of the property estimates helped stabilize the inversion in iteration regions with significant property updates, typically within the first 25% of the iterative process. This refinement was applied to the shear and tensile anisotropy, which are more challenging to estimate than the shear modulus. Throughout the process, the supporting mesh for the shear modulus was kept at a constant isotropic resolution of 1 mm.

## 1.3. Convergence criteria for the TI-NLI algorithm.

Two termination conditions were defined: a minimum size for the property update and a maximum number of iterations. The minimum size of the property update was set to a negligibly small value to ensure that the prescribed maximum number of iterations became the preferred termination criterion. Convergence was evaluated by monitoring the average property values in the computational domain over the iterations. The reconstruction was deemed converged when the curve stabilized.

Convergence criteria are now detailed in **Table S.1,**

**Figure 4** in the main manuscript presents the aMRE workflow, and the iteration steps are detailed in page 13 after the definition of bulk modulus.

*Table S.1 NLI Reconstruction Parameters.*

| Real shear modulus $\mu_{R}$ | 2 kPa |
| --- | --- |
| Material property mesh resolutions | 5mm, 2mm and 1mm |
| Zone sizes (not including overlap factor) | 50 mm |
| Overlap factor for each (x, y, z) direction | 15% |
| Total iterations | 400 |
| Iteration Limits for Zone Iteration Structures | 10,70 |
| Zone Iteration Structures | Conjugate gradient method \| # of line search; 2 \| 2; 3 \| 2; 4 \| 2 |
| Damping ratio | 0.10 |
| $\rho$ (density) | 1.0 g/cm³ |
| $\nu$ (Poisson’s ratio) | 0.499 |
| Gaussian smoothing at each iteration | 1.5 mm kernel width |
| [Lower: Upper] bounds of $\mu$ | [200 Pa: 500 kPa] |
| [Lower: Upper] bounds of $\phi$ and $\zeta$ | [-5:5] for $\phi$ and [-5:5] for $\zeta$ |
| $\phi$ and $\zeta$ initial estimates | $\phi=1$ and $\zeta=1$ for the fiber model  $\phi=-0.5$ and $\zeta=-0.5$ for the sheet model |

# 2. Results

## 2.1. Damping ratio

Displacement attenuation patterns, characterized by the damping ratio $\xi=\frac{Im\left[ \mu_{tra} \right]}{2Re[\mu_{tra}]}$, were different in each specimen but showed some visual correspondence with the measured OSS-SNR in the mappings (**Figure S.1**). Some specimens (#3, #4, and #9) had highly damped septum ($\xi$ >1), while others did not ($0<\xi<0.6$), indicating that oscillations decay very rapidly without oscillating in these cases, dissipating energy due to internal friction or higher viscosity. This can be due to various factors, including the tissue's microstructure including extracellular matrix components, cell density, fluid content, or even pathological changes. The median damping ratio and [$\frac{1}{4}:\frac{3}{4}$] percentiles in the specimens was 0.09 [0.04:0.20] (mean$\pm$SD = 0.18$\pm$0.21) compared to the 0.1 initial value used in TI-NLI based on literature FE studies [2].

*Figure S.1 Octahedral shear strain signal to noise ratio (OSS SNR), blue color scale, and damping ratio, green color scale, for each of the 10 specimens at slice 2 and 2^nd^ repeated acquisition. Damping ratio scale is variable to highlight subtle differences within each specimen.*

## 2.2. Wave displacement and error

Amplitude and phase were quantified with error below 20% across directions, specimens, and repetitions (${error}_{x}=19.4\pm18.5\%, {error}_{y}=19.5\pm18.7\%, and {error}_{z}= 19.6\pm19.0\%$) (**Figure S.2**). For all experiments, convergence of MRE metrics were reached regardless of the specimen. Convergence plots of $\mu_{tra}$, $\phi$, and $\zeta$ are shown in **Figure S.3, A.4**, **A.5, and A.6** for each model.

*Figure S.2 Amplitude, phase and error in (x, y, z) directions for specimen #1*

## 2.3. Convergence

*Figure S.3 Convergence plots of* $\mu_{tra}$*, real and imaginary parts, using the fiber model*

*Figure S.4 Convergence plots of* $\mu_{tra}$*, real and imaginary parts, using the sheet model*

*Figure S.5 Convergence plots of* $\mu_{tra}$*, real and imaginary parts, using the isotropic incompressible model.*

*Figure S.6 Convergence plots of shear anisotropy (*$\phi$*) and tensile anisotropy (*$\zeta$*) over 330 iterations in specimen 1 with the fiber model.*

## 2.4. Variations Between Samples

Anisotropic MRE metrics for each specimen were consistent and within the same range (**Figure S.*7***), except for a few specimens having lower OSSR (specimen #2 and #3), as discussed in the previous sections.

Figure S.7 *MRE metrics for each specimen (#1 to #10) are shown, [1:10] comprised all the values from specimen #1 to #10. The first row shows the axial shear modulus (*$\mu_{ax}$*), the transverse shear modulus (*$\mu_{tra}$)*. The second row shows the axial Young’s modulus (*$E_{ax}$*), the transverse Young’s modulus (*$E_{tra}$*). The third row shows the shear anisotropy (*$\phi$*) and the tensile anisotropy (*$\zeta$*). The first quartiles are indicated by the boxes while the maximum whisker length indicates the interquartile range.*

# 3. Reported myocardial stiffness in the literature

We reported three methods of studying myocardial stiffness with or without considering the anisotropic structure: Finite Element (FE) modelling with or without the fiber field, MR elastography with or without DTI, and mechanical load tests with their respective models (e.g. Holzapfel and Ogden ‘s model) (**Table S.2**).

*Table S.2 Reported myocardial stiffness values [kPa] in the literature in pigs or humans, by MRE, FE or mechanical stretch/tensile load tests. When the mean myocardial stiffness was reported without specifying which modulus, we assumed it was the real shear modulus*${(\mu}_{R}=\mu_{tra})$*.*

| References  (values in [kPa]) | Healthy (vs disease) | | | | | | |
| --- | --- | --- | --- | --- | --- | --- | --- |
| Guccione, 1995, [3]  Simulation, Finite Element Modelling, simulated fiber field | C = 0.876 kPa (Transversely isotropic material) $b_{f}=18.48$ \|; $b_{t}=3.58$ \| $b_{fs}=1.62$; Which results, from the second derivative of the strain energy potential W, $E_{ax}=C.b_{f}=16.18$ $E_{tra}=C.b_{t}=3.13$ $\mu_{ax}=C.b_{fs}=1.42$ $\mu_{tra}=C.b_{sn}$ ***Fiber angle***: [+87.3°: -46.8°] (human, simulated), linear helix \| | | | | | | |
| Dokos, 2002 [4]  Pigs (n=6) | According to [5] with HO method a = 0.073 \| b=15.517 \| Which results in \| $E_{ax}=a_{ff}\left( 1+2a \right)=26.04\left( 1+2*0.073 \right)=29.84$; $a_{ff}=26.04$ \| $b_{f}=9.33$ \| \| $E_{tra}=a_{ss}\left( 1+2a \right)=5.57$; $a_{ss}=4.87$ \| $b_{ss}=0.001$ \| \| $\mu_{ax}=a_{fs}\left( 1+a \right)=0.19$; $a_{nn}=-$ \| $b_{nn}=-$ \| \| $\mu_{tra}=a_{sn}\left( 1+a \right)$; $a_{fs}=0.17$ \| $b_{fs}=16.955$ \| \|; $a_{fn}=-$ \| $b_{fn}=-$ \| \|; $a_{sn}=-$ \| $b_{sn}=-$ \| \| With Holzapfel and Ogden, denoted as the general HO model [5]: a = 0.019 \| b=8.576 \| Which results in \| $E_{ax}=a_{ff}\left( 1+2a \right)=26.77$; $a_{ff}=25.79$ \| $b_{f}=9.67$ \| \| $E_{tra}=a_{ss}\left( 1+2a \right)=4.44$; $a_{ss}=4.28$ \| $b_{ss}=0.001$ \| \| $\mu_{ax}=a_{fs}\left( 1+a \right)=0.26$; $a_{nn}=0.001$ \| $b_{nn}=0.87$ \| \| $\mu_{tra}=a_{sn}\left( 1+a \right)=0.26$; $a_{fs}=0.25$ \| $b_{fs}=16.04$ \| \|; $a_{fn}=0.025$ \| $b_{fn}=0.025$ \| \|; $a_{sn}=0.25$ \| $b_{sn}=13.82$ \| \| However, according to [6] and [5], this “a” value is wrong. | | | | | | |
| Kolipaka, 2010, [7]  pigs (n=6),  MRE (80Hz, 1.04 x 4.21 x 5 mm³, 1 slice),  **no DTI** | $\mu_{Tra}$  ***Fiber angle***: Not accounted (pigs) | | | End systole | | | End diastole |
|  |  |  |  | 9.34±1.9 | | | 6.03±1.8 |
| Kolipaka, 2011, [8]  18 Healthy, 2 HCM  MRE(80Hz, 1.63x5.62x8mm3,2 slices)  **no DTI** | $\mu_{tra}$  ***Fiber angle***: Not accounted (human, HCM) | | | End systole | | | End diastole |
|  |  |  |  | 2.7 (vs 7 in HCM) | | | 5.6 (vs 14.5 in HCM) |
| Kolipaka, 2011, [9],  pigs (n=4),  MRE (80Hz, 1.59x5.46x8mm3, 1 slice),  **no DTI** | $\mu_{tra}$  ***Fiber angle***: Not accounted (pigs) | | |  | | | End diastole |
|  |  |  |  |  | | | 8.9 |
| Elgeti 2014, [10]  Healthy: 10 old, 10 young volunteers  Diastolic dysfunction: 10 old volunteers  MRE (24 Hz, 2.5x2.6x7mm3, 1 slice),  **no DTI** | $\mu_{tra}$  ***Fiber angle:*** Not accounted (human) | | |  | | |  |
|  |  |  |  | Shear wave amplitude ? | | |  |
| Mazumder 2015, [11],  Pigs (n=1),  MRE(80 Hz, 2.5x2.5x2.5mm3, 5-7 slices)  **DTI** (12dir, TE/TR=80ms/3200ms, b=1000 s/mm2, 10 av, 2.5x2.5x2.5mm3,) |  | | | End systole | | | End diastole |
|  | $c_{11}= \kappa+\frac{4}{3}\mu_{tra}\left( 1+\frac{4}{3}\zeta\right)$ | | | 13.97±0.38 | | | 5.49±0.07 |
|  | $c_{22}=c_{33}=$  $\kappa+\frac{4}{3}\mu_{tra}\left( 1+\frac{1}{3}\zeta\right)$ | | | 8.68±0.19 or 27.34±1.02 | | | 7.02±0.22 or 20.00±0.54 |
|  | ${C_{44}=C_{66}=\mu}_{ax}$ | | | 6.54±1.23 or 6.45±0.68 | | | 2.52±0.31 or 5.53±0.31 |
|  | ${C_{55}=\mu}_{tra}$ | | | 6.44±0.62 | | | 5.56±0.39 |
|  | ***Fiber angle:*** not provided (pigs) | | | | | | |
| Mazumder 2017, [12]  Pigs (n=8),  MRE(80 Hz, 3x3x8mm3, 5-7 slices)  **no DTI** | $\mu_{tra}$ | | | 3.84±0.4 (vs 4.82±0.2 in infarct) | | | 4.94±0.5 (vs in 5.88±0.5 in infarct) |
|  | ***Fiber angle:*** Not accounted (pigs) | | | | | | |
| Arani 2017 [13],  11 healthy  16 Amyloidosis  MRE (140 Hz, 5mm isotropic, 5 slices),  **no DTI** |  | | Early systole | | | | |
|  | $\mu_{tra}$ | | 8.2 [7.2: 11.8], median [min:max]  (vs 11.4 [9.2: 15.7] in cardiac amyloidosis) | | | | |
|  | ***Fiber angle:*** Not accounted (human, amyloidosis) | | | | | | |
| Wang, 2013, [14]  Simulation, Finite Element Modelling , simulated fiber field  Also used by Colombo, 2014 [15], | (Holzapfel and Ogden ‘s model) a = 0.2362 kPa \| b = 10.81; $a_{ff}=20.037$ kPa \| $b_{ff}=14.15$; $a_{ss}=3.7245$ kPa \| $b_{ss}=5.16$; $a_{fs}=0.4108$ kPa \| $b_{fs}=11.30$; which results in $E_{ax}=a_{ff}\left( 1+2a \right)=29.49$ $E_{tra}=a_{ss}\left( 1+2a \right)=5.48$ $\mu_{ax}=a_{fs}\left( 1+a \right)=0.60$ $\mu_{tra}=a_{sn}\left( 1+a \right)$ According to Gultekin et al [6], the initial guess of “a” coefficient is overestimated and wrong from Wang et al [14]. ***Fiber angle:*** [+60°: -60°] (human, rule-based simulation), linear helix centered at mid wall (0°) ***Sheet angle***: [-45°: +45°] \| | | | | | | |
| Eriksson 2013, [16],[17]  Simulation, Finite Element Modelling , simulated fiber field | Considered myofibre and sheet dispersion for the two anisotropic terms in the H-O model. a = 0.333 kPa \| b = 9.242; $a_{ff}=18.535$kPa \| $b_{ff}=15.972$; $a_{ss}=2.564$ kPa \| $b_{ss}=10.446$; $a_{fs}=0.417$ kPa \| $b_{fs}=11.602$ Which results in $E_{ax}=a_{ff}\left( 1+2a \right)=30.87$ $E_{tra}=a_{ss}\left( 1+2a \right)=4.27$ $\mu_{ax}=a_{fs}\left( 1+a \right)=0.69$ $\mu_{tra}=a_{sn}\left( 1+a \right)$ ***Fiber angle:*** [+85°: -60°] (human, rule-based simulation), linear helix centered at mid wall (0°) ***Sheet angle***: [-45°: +45°] | | | | | | |
| Sommer, 2015 [18],  Mechanical loading tests, human ex vivo (n=28) | According to [5]: a = 0.809 kPa $a_{ff}=1.911$ kPa \| $a_{fs}=0.547$ kPa; $a_{ss}=-$kPa \| $a_{fn}=-$kPa; $a_{nn}=0.227$ kPa \| $a_{sn}=-$kPa Which, considering Holzapfel and Ogden model, results in $E_{ax}=a_{ff}\left( 1+2a \right)=5.0$ $E_{tra}=a_{ss}\left( 1+2a \right)$ $\mu_{ax}=a_{fs}\left( 1+a \right)=1.43$ $\mu_{tra}=a_{sn}\left( 1+a \right)$ With Holzapfel and Ogden, denoted as the general HO model [5]: a = 0.180 kPa $a_{ff}=2.24$kPa \| $a_{fs}=1.29$ kPa; $a_{ss}=0.098$kPa \| $a_{fn}=1.34$kPa; $a_{nn}=0.508$ kPa \| $a_{sn}=0.94$kPa which results in $E_{ax}=a_{ff}\left( 1+2a \right)=3,04$ $E_{tra}=a_{ss}\left( 1+2a \right)=0.13$ $\mu_{ax}=a_{fs}\left( 1+a \right)=1.75$ $\mu_{tra}=a_{sn}\left( 1+a \right)=1.28$ 14.8±6.9° per mm depth with 13±4 mm septum thickness => calculated ***Fiber angle:*** (14.8*13/2) =>[-96°: +96°] (human) linear helix centered at mid wall (0°) | | | | | | |
| Gultekin 2016, [6]  FEM, simulated fiber field from DTI data from [19] | Biaxial extension testing  a = 1.36 kPa, b = 21.75  $a_{f}$ = 0.49 kPa, $b_{f}$ = 90.10  Which, considering orthotropic viscoelastic model, results in  $E_{ax}=a_{f}=0.49$  $E_{tra}=a_{s}$  $\mu_{ax}=a_{fs}$  $\mu_{tra}=a_{sn}$  Triaxial shear testing  a = 0.40 kPa, b = 6.55  $a_{f}$ = 3.05 kPa, $b_{f}$ = 29.05  $a_{s}$ = 1.25 kPa, $b_{s}$ = 36.65  $a_{fs}$ = 0.15 kPa, $b_{fs}$ = 6.28  Which, considering orthotropic viscoelastic model, results in  $E_{ax}=a_{f}=3.05$  $E_{tra}=a_{s}=1.25$  $\mu_{ax}=a_{fs}=0.15$  $\mu_{tra}=a_{sn}$  ***Fiber angle:*** [-60°: +40°], septum segment (human, ex vivo, simulated). [-20°: +50°], lateral segment from [19], Linear helix | | | | | | |
| Wassenaar 2016, [20],  29 healthy  MRE (80 Hz, 1.56x6.25x10mm3, 5 slices), **no DTI** | $\mu_{tra}$  ***Fiber angle:*** not accounted (human) | | End systole | | End diastole | | |
|  |  |  | 6.10±1.38 | | 4.99±1.05 | | |
| Arunachalam 2017 [21]  Pigs (n=5)  MRE (140 Hz, 3x3x3mm3, 1 slice), **no DTI** | $\mu_{tra}$  ***Fiber angle:*** not accounted (human) | |  | | Early diastole | | |
|  |  |  |  | | 3.0± 0.6 (vs 4.6± 0.7 in infarct) | | |
| Arunachalam 2017 [22]  Pigs (n=17)  MRE (140 Hz, 3x3x3mm3, 1 slice), **no DTI** | $\mu_{tra}$  ***Fiber angle:*** not accounted (human) | | End systole | | End diastole | | |
|  |  |  | 5.89±0.34 | | 3.91±0.39 | | |
| Miller 2017 [2],  Simulation, Finite Element Modelling **with simulated DTI** and MRE at 80Hz | $E_{tra}=E1 =E2= 6.53 \pm0.05 kPa,$(Transverse Young’s moduli)  $E_{ax}=E3 = 11.03\pm0.63 kPa$, (Fibre Young’s modulus)  $\mu_{ax}=G_{13}=G_{23}= 2.53\pm0.05 kPa$(Fibre shear moduli)  $v_{12}=v_{31}=v_{32}= 0.49999999$(Fibre and Transverse Poisson’s ratio)  dc=0.1 (structural damping coefficient)  $\rho=1.2 g/cm^{3}$ (density) (initially set at 1.06 $g/cm^{3}$)  ***Fiber angle:*** [-60°: +60°], (human, simulated)  linear helix centered at mid wall (0°) | | | | | | |
| Miller 2018 [23],  Simulation, Finite Element Modelling **with simulated DTI** | $E_{tra}=E1 =E2= 6.59 \pm0.24 kPa,$(Transverse Young’s moduli)  $E_{ax}=E3 = 10.80 \pm0.48 kPa$, (Fibre Young’s modulus)  $\mu_{tra}=G_{12}= 1.94 \pm0.07 kPa$, (Transverse shear modulus)  $\mu_{ax}=G_{13}=G_{23}= 2.52 \pm0.05 kPa$(Fibre shear moduli)  $v_{31}=v_{32} = 0.4999$ (Fibre Poisson’s ratio)  $v_{12}= 0.4999$ (Transverse Poisson’s ratio)  $\tau=3.1056 KPa$ (Anisotropic tensile ratio)  $\kappa=3.2500 e4 KPa$ (Bulk modulus)  ***Fiber angle:*** [-60°: +60°], (human, simulated)  linear helix centered at mid wall (0°) | | | | | | |
| Wang, 2018 [24]  FEM, simulated fiber field from [25] | $\mu_{tra}$ |  | | | | End diastole | |
|  |  |  | | | | 1.2+-0.4 (vs 2.7+-0.6 in HFpEF) | |
|  | ***Fiber angle:*** [-60°: +60°], lateral segment (human, simulated) from [25], [-70°: +70°], septum, sigmoidal helix | | | | | | |
| Ahmad, 2018 [26]  FEM, simulated fiber field | According to [5]: a = 0.075 $a_{ff}=7.06$ \| $a_{fs}=1.86$; $a_{ss}=-$ \| $a_{fn}=3.54$; $a_{nn}=2.74$ \| $a_{sn}=-$ Which, considering Holzapfel and Ogden model, results in $E_{ax}=a_{ff}\left( 1+2a \right)=8.11$ $E_{tra}=a_{ss}\left( 1+2a \right)$ $\mu_{ax}=a_{fs}\left( 1+a \right)=2.13$ $\mu_{tra}=a_{sn}\left( 1+a \right)$ With Holzapfel and Ogden, denoted as the general HO model [5]: a = 0.005 $a_{ff}=7.21$ \| $a_{fs}=1.07$; $a_{ss}=2.24$ \| $a_{fn}=3.34$; $a_{nn}=3.22$ \| $a_{sn}=0.42$; Which results in $E_{ax}=a_{ff}\left( 1+2a \right)=7.28$ $E_{tra}=a_{ss}\left( 1+2a \right)=2.26$ $\mu_{ax}=a_{fs}\left( 1+a \right)=1.08$ $\mu_{tra}=a_{sn}\left( 1+a \right)=0.42$ \| | | | | | | |
| Guan, 2019 [5]  Simulation, Finite Element Modelling, simulated fiber field | Preferred HO-D results. With Holzapfel and Ogden, denoted as the general HO model [5]: a = 0.019 $a_{ff}=25.79$ \| $a_{fs}=0.25$; $a_{ss}=4.28$ \| $a_{fn}=0.025$; $a_{nn}=0.001$ \| $a_{sn}=0.252$ Which results in $E_{ax}=a_{ff}\left( 1+2a \right)=26.77$ $E_{tra}=a_{ss}\left( 1+2a \right)=4.44$ $\mu_{ax}=a_{fs}\left( 1+a \right)=0.26$ $\mu_{tra}=a_{sn}\left( 1+a \right)=0.26$ ***Fiber angle:*** [-60°: +60°], (human, simulated) linear helix centered at mid wall (0°) | | | | | | |
| Li, 2019, [27]  FEM, acquired DTI | (Orthotropic, Holzapfel and Ogden ‘s model) \| a (kPa) \| b \| $a_{f}$ (kPa) \| $b_{f}$ \| $a_{s}$ (kPa) \| $b_{s}$; S1 \| 0.00481 \| 5.05 \| 1.17 \| 0.276 \| 1.66 \| 6.93; S4 \| 0.00105 \| 12.0 \| 2.96 \| 3.10 \| 0.50 \| 0.0870 \| $a_{fs}$ (kPa) \| $b_{fs}$ \| $a_{fn}$ (kPa) \| $b_{fn}$ \| $a_{sn}$ (kPa) \| $b_{sn}$; S1 \| 0.13 \| 0.024 \| 3.44 \| 2.01 \| 3.28 \| 20.4; S4 \| 0.37 \| 0.011 \| 1.71 \| 68.8 \| 0.545 \| 1.17 Which, considering orthotropic viscoelastic model, results in \| $E_{ax}={2ab+a}_{f}b_{f}$ \| $E_{tra}={2ab+a}_{s}b_{s}$ \| $\mu_{tra}={2a+a}_{fs}$ \| $\mu_{ax}={2a+a}_{sn}$; S1 \| 0.37 \| 11.55 \| 1.67 \| 3.28; S4 \| 9.20 \| 0.06 \| 0.50 \| 0.54 ***Fiber angle:*** (human, simulated) Where S1 has helical fibers going from 0° at epicardium to 90° at endocardium Where S4 has helical fibers going from -45° at epicardium to +45° at endocardium | | | | | | |
| Rumindo 2020 [28] FEM, simulated fiber field  Human | $\mu_{tra}=$1.60 ± 0.22 C (kPa) \| $b_{f}$ \| $b_{t}$ \| $b_{ft}$; 0.08 \| 16.15 \| 6.46 \| 11.31 Which results, from Payan and Ohayon [29] , in $E_{ax}=\frac{C}{2}\left( 2b_{f}+b_{t} \right)=1.55$ $E_{tra}=\frac{Cb_{t}\left( 2b_{f}+b_{t} \right)}{b_{f}+b_{t}}=0.88$ ***Fiber angle:*** [ -52°:+74°] (human, simulated) linear helix centered at mid wall (0°) | | | | | | |
| Guan, 2022, [30] | Focus on how fibre dispersion affects myocardial passive behaviours, in particular the sheet dispersion, with calibrated H-O model. Case \| a (kPa) \| b \| $a_{f}$ (kPa) \| $b_{f}$ \| $a_{s}$ (kPa) \| $b_{s}$; 1 \| 0.50 \| 9.69 \| 4.23 \| 0.92 \| 0.006 \| 0.042; 3 \| 1.06 \| 5.87 \| 1.87 \| 7.64 \| 0.107 \| 13.43 Case \| $a_{fs}$ (kPa) \| $b_{fs}$; 1 \| 0.61 \| 5.74; 3 \| 0.23 \| 0.001 Which results with H-O model in case \| $E_{ax}=a_{ff}\left( 1+2a \right)$ \| $E_{tra}=a_{ss}\left( 1+2a \right)$ \| $\mu_{ax}=a_{sn}\left( 1+a \right)$ \| $\mu_{tra}=a_{fs}\left( 1+a \right)$; 1 \| 8.46 \| 0.01 \| NA \| 1.22; 3 \| 5.88 \| 0.34 \| NA \| 0.72 14.8° per mm depth with 8.7 mm LV thickness => calculated ***Fiber angle:*** [-60°: +60°] (human) linear helix centered at mid wall (0°) Case 3 considers sheet dispersion. | | | | | | |

*Table S.3 Young’s modulus [kPa] at stretch level 1.075 and 1.1 obtained from equibiaxial extension testing in* ***humans*** [18]*.*

| Stretches | Human (n=28) | | | | | | | | | | | |
| --- | --- | --- | --- | --- | --- | --- | --- | --- | --- | --- | --- | --- |
|  | LV | |  | LVFW | |  | Septum | |  | RVFW | | |
|  | $E_{ax}$ | $E_{tra}$ |  | $E_{ax}$ | $E_{tra}$ |  | $E_{ax}$ | $E_{tra}$ |  | $E_{ax}$ | $E_{tra}$ |  |
| 1.075 | 68±34 | 37±17 |  | 70±38 | 34±16 |  | 61±21 | 45±20 |  | 85±22 | 64±8 |  |
|  | (n=31) | |  | (n=24) | |  | (n=7) | |  | (n=3) | | |
| 1.1 | 83±23 | 49±23 |  | 79±30 | 47±22 |  | 85±19 | 53±26 |  | 141±19 | 108±6 |  |
|  | (n=26) | |  | (n=19) | |  | (n=7) | |  | (n=3) | | |
| 1.125 | 97±32 | 47±14 |  | 91±33 | 48±14 |  | 108±30 | 44±13 |  | 278 | 205 |  |
|  | (n=20) | |  | (n=15) | |  | (n=5) | |  | (n=2) | | |

*Table S.4 Young’s modulus [kPa] at stretch level 1.15 obtained from equibiaxial extension testing in* ***humans*** [31]

| Stretches | Human (n=8) | |
| --- | --- | --- |
|  | LV | |
|  | $E_{ax}$ | $E_{tra}$ |
| 1.15 | 140±56 | 16.3±6.1 |

*Table S.5 Shear modulus [kPa] obtained from triaxial shear testing of human left ventricular myocardial cubes (LVFW, septum) in* ***humans*** [18]*. In (*), half of the peak to peak stress for a given shear displacement is reported* [4]*.*

| Amount of shear [KPa] | Human (n=28) in the LVFW and Septum | | | | | | |
| --- | --- | --- | --- | --- | --- | --- | --- |
|  | $\mu_{ax}$ | | | |  | $\mu_{tra}$ | |
|  | FS | SF | FN | NF |  | SN | NS |
| 0.2 | 10±3.5 | 8.0±3.0 | 10±3.0 | 7.0±1.5 |  | 7.5±3.0 | 6.5±2.0 |
| 0.4 | 11.5±2.5 | 7.25±3.0 | 10.5±3.25 | 5.75±1.5 |  | 7.0±3.0 | 5.5±2.0 |
| 0.5* | ~24 | ~11.0 | ~20 | ~6.0 |  | ~7.0 | ~5.0 |
| 0.5 | 11.6±3.4 | 7.0±2.4 | 10.8±3.0 | 5.4±2.8 |  | 7.0±2.6 | 5.6±2.0 |

*Table S.6 Shear and Young’s moduli [kPa] retrieved from Fung strain energy coefficients (axial and transversal) measured with mechanical loading in* ***humans*** [31] *for small strain.*

| References | myocardium | Amount of shear | Shear modulus | | Young’s modulus | |
| --- | --- | --- | --- | --- | --- | --- |
|  |  |  | $\mu_{ax}=C.b4=C.b6$ | $\mu_{tra}=C.b5$ | $E_{ax}=C.b1$ | $E_{tra}=C.b2$ |
| Fatemifar, [31],  n = 8 | LV | 0.4 | 13±3.25 | NA | 45±6 | 22.5±4 |
|  | LV papillary muscles | 0.4 | 11.25±0.5 | NA | 66±6 | 47±4.5 |
|  | LV trabeculae | 0.4 | NA | NA | 80±7 | NA |
|  | RV | 0.4 | 5±0 | NA | 50±0.5 | 40±1.5 |
|  | RV papillary muscles | 0.4 | 5±0.25 | NA | 75±0.5 | 31±2.5 |
|  | RV trabeculae | 0.4 | NA | NA | 12±3 | NA |

*Table S.7 Young’s moduli [kPa] (axial and transversal) measured with mechanical loading in* ***sheep and porcine neonates***

| References | myocardium | Stretches  (tension) | $E_{ax}=\frac{stress_{normal}}{strain_{normal}}$ (MFD) | $E_{tra}=\frac{stress_{normal}}{strain_{normal}}$  (CFD) | |
| --- | --- | --- | --- | --- | --- |
|  |  |  | FF | SS | NN |
| Kakaletsis [32],  (sheep, n=12) | RV | 0.094 | ~19.15±5.32 | ~7.45±3.19 | ~3.19±1.06 |
| Ahmad[26]  (pig neonates, n=43) | RVFW | failure stress (0.5 baseline) | 16.22±4.3 | 9.38±2.8 | 16.44±3.6 |
|  | LVFW |  | 13.94±1.8 | 11.68±0.8 | 11.9±0.8 |

*Table S.8 Shear modulus [kPa] (axial and transversal) measured with mechanical loading in* ***sheep and porcine neonates****.*

| References | myocardium | Amount of shear | $\mu_{ax}$ (MFD) | | | | $\mu_{tra}$ (CFD) | |
| --- | --- | --- | --- | --- | --- | --- | --- | --- |
|  |  |  | FS | SF | FN | NF | SN | NS |
| Kakaletsis [32],  (sheep, n = 12) | RV | 0.282 | ~3.55±1.06 | ~2.84±0.71 | ~2.13±0.71 | ~1.42±0.35 | ~1.77±0.71 | ~2.13±0.71 |
| Ahmad [26]  (pig neonates, n=43) | RV ant. | 0.5 | 6.76±1.0 | | | | 9.24±0.8 | |
|  | RV post. | 0.5 | 3.68±1.0 | | | | 6.44±0.8 | |
|  | LV ant. | 0.5 | 4.20±1.0 | | | | 7.20±0.8 | |
|  | LV post. | 0.5 | 4.50±1.0 | | | | 5.06±0.8 | |

# Summary

*Table S.9 Reported Young's and shear moduli in the literature using Finite Element (FE) simulation*

| FEM | Guccione 1995 | Dokos, 2002  recalculated by Guan 2019 | | Wang, 2013 | Eriksson 2013 | Gultekin 2016 | Miller 2017 | Miller 2018 | Wang, 2018 | Ahmad, 2018 recalculated by Guan 2019 |
| --- | --- | --- | --- | --- | --- | --- | --- | --- | --- | --- |
| type | human | pig | | human | human | human | human | human | human | Pig neonate |
| $E_{ax}$ | $16.18$ | $29.84$ | $26,77$ | $29.49$ | 30.87 | $3.05$ | $11.03$ | $10.80$ |  | $8.11$ |
| $E_{tra}$ | $3.13$ | $5.57$ | $4.44$ | $5.48$ | 4.27 | $1.25$ | $6.53$ | $6.59$ |  |  |
| $\mu_{ax}$ | $1.42$ | $0.19$ | $0.26$ | $0.60$ | 0.69 | $0.15$ | $2.53$ | $2.52$ | 1.2 | $2.13$ |
| $\mu_{tra}$ |  |  | $0.26$ |  |  |  |  | $1.94$ |  |  |

*Table S.10 Reported Young's and shear moduli in the literature using Finite Element (FE) simulation*

| FEM | Guan, 2019 | Li, 2019 | | Rumindo 2020 | Guan, 2022 | | Sommer, 2015 | | Dokos 2002 | Ahmad, 2018 |
| --- | --- | --- | --- | --- | --- | --- | --- | --- | --- | --- |
|  |  |  |  |  |  |  | Recalculated by Guan 2019 | | | |
|  | human | human | | human | human | | human | | pig | Pig neonates |
| $E_{ax}$ | $26.77$ | 0.37 | 9.20 | 1.55 | 8.46 | 5.88 | $5.0$ | $3,04$ |  | 7.28 |
| $E_{tra}$ | $4.44$ | 11.55 | 0.06 | 0.88 | 0.01 | 0.34 |  | $0.13$ |  | 2.26 |
| $\mu_{ax}$ | $0.26$ | 3.28 | 0.54 |  |  |  |  | $1.28$ | 24 | 1.08 |
| $\mu_{tra}$ | $0.26$ | 1.67 | 0.50 | 1.60 | 1.22 | 0.72 | $1.43$ | $1.75$ | 7 | 0.42 |

*Table S.11 Reported shear moduli in the literature using MRE.*

| MRE | Kolipaka, 2010 | Kolipaka, 2011 | Kolipaka, 2011 | Mazumder 2015 | Mazumder 2017 | Arani 2017 | Wassenaar 2016 | Arunachalam 2017 | Arunachalam 2017 |
| --- | --- | --- | --- | --- | --- | --- | --- | --- | --- |
| type | pig | human | pig | Human | pig | human | human | pig | pig |
| $\mu_{tra}$ | 6.03 | 5.6 | 8.9 | 5.56 | 4.94 | 8.2 systole | 4.99 | 3.0 | 3.91 |
| $\mu_{ax}$ |  |  |  | 2.52 |  |  |  |  |  |

*Table S.12 Reported Young's and shear moduli in the literature using equibiaxial tension test or triaxial shear tests.*

| Mechanical | Sommer et al., 2015 | Ahmad 2018 | Fatemifar et al., 2019 | | Kakaletsis 2021  (RV) |
| --- | --- | --- | --- | --- | --- |
|  |  |  |  |  |  |
| type | human | Pig neonates | human | | sheep |
| $E_{ax}$ | 97±32 | 13.94±1.8 | 140±56 | 45±6 | 19.15±5.32 |
| $E_{tra}$ | 47±14 | 11.68±0.8 | 16.3±6.1 | 22.5±4 | 7.45±3.19 |
| $\mu_{ax}$ | 11.6±3.4 | 4.20±1.0 |  | 13±3.25 | 3.55±1.06 |
| $\mu_{tra}$ |  | 7.20±0.8 |  |  | 1.77±0.71 |

Figure S.8 Detailed reference frame for each shear modulus and corresponding shear deformation modes. The fiber model (A) and sheet model (B) with their respective axial ($\mu_{ax}$) and transverse ($\mu_{Tra}$) shear moduli and corresponding deformation modes. In 1) and 2) The eigenvectors ($\vec{E_{1}},\vec{E_{2}} and \vec{E_{3}}$) refer to the diffusion tensor eigenvectors. Colored arrows depict the direction of shearing, sheets are rectangular prisms and fiber are cylinders. In 3) and 4) dotted lines represent the resistance to the shear modes along the fiber axis(F), along the sheet axis (S) and normal to sheet plane(N).

Figure S.9 Detailed reference frame for each Young’s modulus and corresponding tensile deformation modes. The fiber model (A) and sheet model (B) with their respective transverse ($E_{Tra}$ in 1) ) and axial ($E_{ax}$ in 2) ) Young’s moduli and corresponding deformation modes. Colored arrows depict the direction of stretching, sheets are rectangular prisms and fiber are cylinders. Tensile modes are shown respectively to the fiber (F), sheet (S) and normal to the sheet (N) planes, as red arrows depict direction of stretching. FF, SS, and NN are the conventional stretching directions from mechanical loading studies (hysteresis curves).

# Bibliography

[1]	E. Barnhill, P. Kennedy, C. L. Johnson, M. Mada, and N. Roberts, “Real-time 4D phase unwrapping applied to magnetic resonance elastography,” *Magn. Reson. Med.*, vol. 73, no. 6, pp. 2321–2331, Jun. 2015, doi: 10.1002/MRM.25332/ASSET/SUPINFO/MRM25332-SUP-0001-SUPPINFO01.PDF.

[2]	R. Miller, A. Kolipaka, M. P. Nash, and A. A. Young, “Relative identifiability of anisotropic properties from magnetic resonance elastography,” *NMR Biomed.*, vol. 31, no. 10, p. e3848, Oct. 2018, doi: 10.1002/NBM.3848.

[3]	J. M. Guccione, K. D. Costa, and A. D. McCulloch, “Finite element stress analysis of left ventricular mechanics in the beating dog heart,” *J. Biomech.*, vol. 28, no. 10, pp. 1167–1177, Oct. 1995, doi: 10.1016/0021-9290(94)00174-3.

[4]	S. Dokos, B. H. Smaill, A. a Young, and I. J. LeGrice, “Shear properties of passive ventricular myocardium.,” *Am. J. Physiol. Heart Circ. Physiol.*, vol. 283, no. 6, pp. H2650–H2659, Dec. 2002, doi: 10.1152/ajpheart.00111.2002.

[5]	D. Guan, F. Ahmad, P. Theobald, S. Soe, X. Luo, and H. Gao, “On the AIC-based model reduction for the general Holzapfel–Ogden myocardial constitutive law,” *Biomech. Model. Mechanobiol.*, vol. 18, no. 4, pp. 1213–1232, Aug. 2019, doi: 10.1007/S10237-019-01140-6/FIGURE S.S/13.

[6]	O. Gültekin, G. Sommer, and G. A. Holzapfel, “An orthotropic viscoelastic model for the passive myocardium: continuum basis and numerical treatment,” *Comput. Methods Biomech. Biomed. Engin.*, vol. 19, no. 15, pp. 1647–1664, Nov. 2016, doi: 10.1080/10255842.2016.1176155.

[7]	A. Kolipaka, P. A. Araoz, K. P. McGee, A. Manduca, and R. L. Ehman, “Magnetic resonance elastography as a method for the assessment of effective myocardial stiffness throughout the cardiac cycle,” *Magn. Reson. Med.*, vol. 64, no. 3, pp. 862–870, Sep. 2010, doi: 10.1002/MRM.22467.

[8]	Kolipaka, “A Feasibility Study: MR Elastography as a Method to Compare Stiffness Estimates in Hypertrophic Obstructive Cardiomyopathy & in Normal Volunteers,” 2011. [Online]. Available: https://archive.ismrm.org/2011/0274.html. [Accessed: 05-Jan-2023].

[9]	A. Kolipaka, K. P. McGee, A. Manduca, N. Anavekar, R. L. Ehman, and P. A. Araoz, “In vivo assessment of MR elastography-derived effective end-diastolic myocardial stiffness under different loading conditions,” *J. Magn. Reson. Imaging*, vol. 33, no. 5, pp. 1224–1228, May 2011, doi: 10.1002/JMRI.22531.

[10]	T. Elgeti, F. Knebel, R. Hättasch, B. Hamm, J. Braun, and I. Sack, “Shear-wave Amplitudes Measured with Cardiac MR Elastography for Diagnosis of Diastolic Dysfunction,” *https://doi.org/10.1148/radiol.13131605*, vol. 271, no. 3, pp. 681–687, Jan. 2014, doi: 10.1148/RADIOL.13131605.

[11]	R. Mazumder, B. D. Clymer, R. D. White, A. Romano, and A. Kolipaka, “In-vivo waveguide cardiac magnetic resonance elastography,” *J. Cardiovasc. Magn. Reson.*, vol. 17, no. S1, p. P35, Dec. 2015, doi: 10.1186/1532-429X-17-S1-P35.

[12]	R. Mazumder, S. Schroeder, X. Mo, B. D. Clymer, R. D. White, and A. Kolipaka, “In vivo quantification of myocardial stiffness in hypertensive porcine hearts using MR elastography,” *J. Magn. Reson. Imaging*, vol. 45, no. 3, pp. 813–820, Mar. 2017, doi: 10.1002/jmri.25423.

[13]	A. Arani *et al.*, “Cardiac MR elastography for quantitative assessment of elevated myocardial stiffness in cardiac amyloidosis,” *J. Magn. Reson. Imaging*, vol. 46, no. 5, pp. 1361–1367, Nov. 2017, doi: 10.1002/JMRI.25678.

[14]	H. M. Wang *et al.*, “Structure-based finite strain modelling of the human left ventricle in diastole,” *Int. j. numer. method. biomed. eng.*, vol. 29, no. 1, pp. 83–103, Jan. 2013, doi: 10.1002/CNM.2497.

[15]	N. M. Colombo, “Numerical modelling of ventricular mechanics : role of the myofibre architecture,” 2014.

[16]	T. S. E. Eriksson, A. J. Prassl, G. Plank, and G. A. Holzapfel, “Influence of myocardial fiber/sheet orientations on left ventricular mechanical contraction,” *Math. Mech. Solids*, vol. 18, no. 6, pp. 592–606, May 2013, doi: 10.1177/1081286513485779.

[17]	T. S. E. Eriksson, A. J. Prassl, G. Plank, and G. A. Holzapfel, “Modeling the dispersion in electromechanically coupled myocardium,” *Int. j. numer. method. biomed. eng.*, vol. 29, no. 11, pp. 1267–1284, Nov. 2013, doi: 10.1002/cnm.2575.

[18]	G. Sommer *et al.*, “Biomechanical properties and microstructure of human ventricular myocardium,” *Acta Biomater.*, vol. 24, pp. 172–192, Sep. 2015, doi: 10.1016/J.ACTBIO.2015.06.031.

[19]	D. Rohmer, A. Sitek, and G. T. Gullberg, “Reconstruction and visualization of fiber and laminar structure in the normal human heart from ex vivo diffusion tensor magnetic resonance imaging (DTMRI) data.,” *Invest. Radiol.*, vol. 42, no. 11, pp. 777–789, Nov. 2007, doi: 10.1097/RLI.0b013e3181238330.

[20]	P. A. Wassenaar *et al.*, “Measuring age-dependent myocardial stiffness across the cardiac cycle using MR elastography: A reproducibility study,” *Magn. Reson. Med.*, vol. 75, no. 4, pp. 1586–1593, Apr. 2016, doi: 10.1002/MRM.25760.

[21]	S. P. Arunachalam *et al.*, “Regional assessment of in vivo myocardial stiffness using 3D magnetic resonance elastography in a porcine model of myocardial infarction,” *Magn. Reson. Med.*, vol. 79, no. 1, pp. 361–369, Jan. 2018, doi: 10.1002/MRM.26695.

[22]	S. P. Arunachalam, A. Arani, K. J. Glaser, R. L. Ehman, and P. A. Araoz, “In vivo assessment of systolic and diastolic myocardial stiffness in pigs using 3d cardiac magnetic resonance elastography,” 2017.

[23]	R. Miller, A. Kolipaka, M. P. Nash, and A. A. Young, “Estimation of transversely isotropic material properties from magnetic resonance elastography using the optimised virtual fields method,” *Int. j. numer. method. biomed. eng.*, vol. 34, no. 6, p. e2979, Jun. 2018, doi: 10.1002/CNM.2979.

[24]	Z. J. Wang, V. Y. Wang, C. P. Bradley, M. P. Nash, A. A. Young, and J. J. Cao, “Left Ventricular Diastolic Myocardial Stiffness and End-Diastolic Myofibre Stress in Human Heart Failure Using Personalised Biomechanical Analysis,” *J. Cardiovasc. Transl. Res.*, vol. 11, no. 4, pp. 346–356, Aug. 2018, doi: 10.1007/S12265-018-9816-Y/METRICS.

[25]	P. M. Nielsen, I. J. Le Grice, B. H. Smaill, and P. J. Hunter, “Mathematical model of geometry and fibrous structure of the heart.,” *Am. J. Physiol.*, vol. 260, no. 4 Pt 2, pp. H1365-78, Apr. 1991, doi: Animals Dogs Endocardium/anatomy & histology Heart/*anatomy & histology Heart Ventricles/anatomy & histology Mathematics *Models, Structural Research Support, Non-U.S. Gov’t.

[26]	F. Ahmad *et al.*, “Biomechanical properties and microstructure of neonatal porcine ventricles,” *J. Mech. Behav. Biomed. Mater.*, vol. 88, pp. 18–28, Dec. 2018, doi: 10.1016/J.JMBBM.2018.07.038.

[27]	D. S. Li *et al.*, “Insights into the passive mechanical behavior of left ventricular myocardium using a robust constitutive model based on full 3D kinematics,” *J. Mech. Behav. Biomed. Mater.*, vol. 103, p. 103508, Mar. 2020, doi: 10.1016/J.JMBBM.2019.103508.

[28]	G. K. Rumindo, J. Ohayon, P. Croisille, and P. Clarysse, “In vivo estimation of normal left ventricular stiffness and contractility based on routine cine MR acquisition,” *Med. Eng. Phys.*, vol. 85, pp. 16–26, Nov. 2020, doi: 10.1016/J.MEDENGPHY.2020.09.003.

[29]	C. W. J. Oomens, M. van Vijven, and G. W. M. Peters, *Biomechanics of Living Organs: Hyperelastic Constitu tive Laws for Finite Element Modeling*. Elsevier Inc., 2017.

[30]	D. Guan *et al.*, “Effects of dispersed fibres in myocardial mechanics, Part I: passive response,” *Math. Biosci. Eng. 2022 43972*, vol. 19, no. 4, pp. 3972–3993, 2022, doi: 10.3934/MBE.2022183.

[31]	F. Fatemifar, M. D. Feldman, M. Oglesby, and H.-C. Han, “Comparison of Biomechanical Properties and Microstructure of Trabeculae Carneae, Papillary Muscles, and Myocardium in the Human Heart,” *J Biomech Eng*, vol. 141(2):021, 2019, doi: 10.1115/1.4041966.

[32]	S. Kakaletsis *et al.*, “Right ventricular myocardial mechanics: Multi-modal deformation, microstructure, modeling, and comparison to the left ventricle,” *Acta Biomater.*, vol. 123, pp. 154–166, Mar. 2021, doi: 10.1016/J.ACTBIO.2020.12.006.
